# Development of a Novel PCB-Degrading Biofilm Enriched Biochar Encapsulated with Sol–Gel: A Protective Layer to Sustain Biodegradation Activity

**DOI:** 10.1021/acsestengg.4c00718

**Published:** 2025-03-06

**Authors:** Qin Dong, Timothy E. Mattes, Gregory H. LeFevre

**Affiliations:** † Department of Civil and Environmental Engineering, University of Iowa, 4105 Seamans Center, Iowa City, Iowa 52242, United States; ‡ IIHR—Hydroscience and Engineering, University of Iowa, 100 C. Maxwell Stanley Hydraulics Laboratory, Iowa City, Iowa 52242, United States

**Keywords:** sol−gel, encapsulation, biofilm integrity, bioremediation technology, PCBs

## Abstract

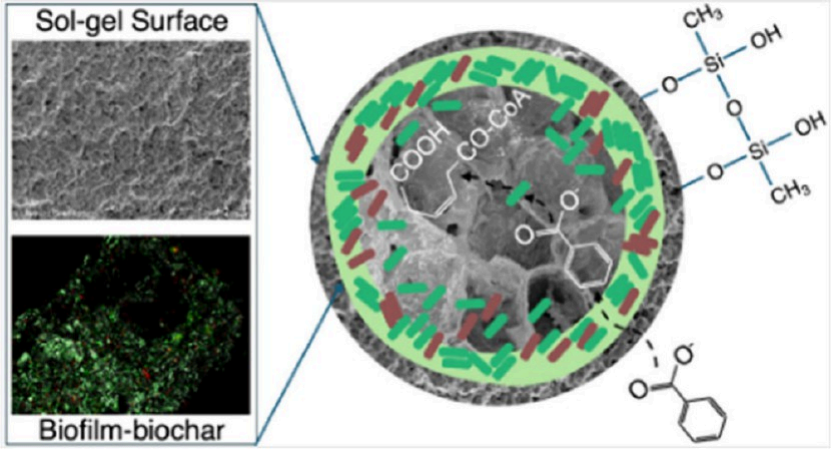

*Paraburkholderia xenovorans* LB400 biofilms hold the potential to degrade PCBs in contaminated sediment. Nevertheless, unfavorable environmental conditions (e.g., salinity, temperature, and shear force) can interfere with biofilm stability and affect biodegradation potential. Sol–gel encapsulation has been used to protect planktonic cell function due to high material stability and absence of cell washout but has not been employed for biofilm protection. Our study is the first to develop sol–gel application on biofilm-enriched black carbons and evaluate efficacy for prolonging biodegradation potential. We systematically tuned multiple sol–gel recipes to coat biofilms and measured the impact of the sol–gel coating on cell survival and pollutant degradation. The developed sol–gel completely encapsulated biofilm-enriched black carbons and produced both high porosity and appropriate pore size that allowed pollutant transfer from the surrounding environment to the biofilms. The sol–gel maintained physical integrity under saline conditions (simulating marine and estuary sediments) and continuously applied shear force. Additionally, the encapsulated biofilms degraded benzoate, a proof-of-concept organic molecule, and extended biofilm attachment and cell viability for over three months without a carbon and energy source. Our study demonstrates that sol–gel helps sustain PCB-degrading biofilms under environmentally relevant conditions. This novel sol–gel application can potentially improve the bioaugmentation effectiveness and enhance degradation of environmental pollutants.

Polychlorinated biphenyls (PCBs) have accumulated and persisted to high levels in sediments decades after being banned. Sediment PCBs can bioaccumulate through food webs and/or expose nearby communities through inhalation^1−5^—an increasingly recognized exposure route^6,7^—which causes adverse human health effects.^8−10^ Lower chlorinated PCBs (LC-PCBs) are more volatile PCBs and can transport from sediment into overlaying water and air;^7^ indeed, dredging does not degrade PCBs and can mobilize sediment LC-PCBs.^11−13^ Anaerobic reductive dechlorination of higher chlorinated PCBs can also contribute to continuous LC-PCB emissions from contaminated sediment.^14^ LC-PCB emissions can be mitigated through bioaugmentation of aerobic microorganisms.^15^ Biofilms grown on black carbons (BCs) are effective in sustaining aerobic PCB-degraders’ viability and inducing PCB biodegradation activity, while in contrast suspended cells are less active.^16^ Nevertheless, the biomass abundance of bioaugmented PCB-degraders with BCs decreases over time following deployment for *in situ* PCB sediment remediation.^14,17,18^ Unfavorable environmental conditions, including shear force (e.g., external water force) and salinity, could drive biomass washout and create intense stress that threatens cell survivability and activity.^19^ Therefore, additional biofilm protection is necessary to increase the longevity of the PCB biodegradation activity.

Encapsulating biofilms with sol–gel holds promise for the enhancement of sustained cell viability and promote prolonged degradation activity. The sol–gel matrix is biocompatible and highly porous, providing mechanical strength and chemical stability.^20−22^ These characteristics make sol–gel a robust framework to enable the transport of substrates and protect biomass against environmental changes. Silica gel is increasingly being used to encapsulate viable whole cells and biomolecules (e.g., protein and enzymes),^23−25^ and encapsulation has been shown to increase enzyme activity and bacterial degradation performance.^22,25,26^ Sodium alginate has also been widely used to immobilize cells and some biofilms because of its low toxicity,^27,28^ but alginate can be physically weakened via dissolution due to ion strength changes or microbially biodegraded over extended time periods.^29,30^ A common two-step process is used to produce sol–gel as follows:^31^ a polymer-forming precursor (e.g., TEOS [tetraethyl orthosilicate], sodium silicate) is hydrolyzed in an acid/base catalyst until sol solution reaches hydrolysis and condensation equilibrium;^20,21,24,32,33^ condensation and gelation are accelerated by adding acid/base catalyst, and gel becomes dense and cross-linked with aging. Although sol–gel has been largely applied to inhibit biofilm formation on sol–gel coated surfaces^34,35^ rather than encapsulating and protecting biofilms, the characteristics of the sol–gel hold high potential and feasibility for application on biofilm-enriched BCs and *in situ* environment.

The versatility of sol–gel chemistry allows tuning of coating characteristics (e.g., mechanical properties, gel microstructure, and cell viability) for numerous applications. For example, addition of MTES [methyltriethoxysilane] to TEOS in sol solution can adjust the hydrophobicity of the final gel product and thereby affect mass transport of substrate.^36^ The use of additives, such as glycerol and polyethylene glycol (PEG), and/or the expulsion of cytotoxic hydrolysis byproducts (e.g., alcohol) during encapsulation, increase gel pore size and improve cell viability.^20,37−39^ Polyvinylpyrrolidone (PVP) helps the applied coating layer to adhere better over the hydrophobic core, while avoiding microcracks.^40−42^ Thus, adjustment of sol–gel chemical conditions can tune properties for specific applications. Employing sol–gel to protect biofilms should maintain cell viability; however, this hypothesis must be tested.

The objective of this study was to develop a novel sol–gel encapsulation approach with the long-term design goal of coating aerobic PCB-degrading biofilm-enriched biochar. Our recent work documented that *Paraburkholderia xenovorans* LB400 biofilms were formed on corn kernel biochar surfaces, as evidenced through two exopolysaccharide production and transport pathways, and discovered that BC feedstocks are critical in influencing biofilm formation and gene expression to improve PCB biodegradation potential.^16^ To further advance *in situ* remediation applications, protecting the biofilm against adverse environmental conditions is critical for deployment. We hypothesize that sol–gel encapsulation of biofilm-enriched biochar can be developed and optimized by changing recipe components, and the encapsulation would increase the longevity of the biofilm’s viability and activity by providing bacteria with a favorable environment. To test our hypothesis, we developed and adjusted the sol–gel coating chemical recipe and coating strategies, measured the physical properties (porous structure, observational hardness, and surface coverage) of the sol–gel product, and evaluated the encapsulated biofilm viability and PCB degradation potential. We discovered this novel encapsulation approach protects biofilms against adverse environmental changes (high salinity and continuous shear force) and extended cell viability for over three months without additional carbon sources. Although this study explicitly focuses on PCB-degrading biofilms, the developed sol–gel approach can also serve as a platform technology for a suite of potential bioremediation applications.

## Methods and Materials

### Chemicals

TEOS and MTES liquid solutions were purchased from Acros Organics (Antwerpen, Belgium). Corn kernel biochar was selected to grow biofilms because we previously demonstrated this type of BC improved cell attachment and functional gene expression; details of biochar characteristics can be found in our prior work.^16^ Corn kernel biochar was obtained from Dr. Albert Ratner’s research group at the University of Iowa.^43^ Glycerol and PEG400 were purchased from Sigma-Aldrich (Burlington, MA) and TCI America (Portland, OR), respectively. PVP was purchased from MP Biomedicals (Santa Ana, California).

### Experimental Design

The desired encapsulation outcome is a complete coating, a porous matrix, and robust observational hardness. Complete coating is defined as a smooth sol–gel surface without cracks and complete sol–gel coverage on biofilm/biochar surface, and it aims to protect biofilms/cells from escaping to maintain biomass. A porous matrix could allow efficient mass transfer of nutrients/pollutants between the surrounding environment and inside sol–gel and concurrently decrease cell leaching. High observational hardness helps prevent the damage of the sol–gel coating from shear force, dissolution, and breaking. All of these are designed to have stable sol–gel structures and adequate food/energy supplies and extend cell survival and activity in a real environment.

**Figure 1:**
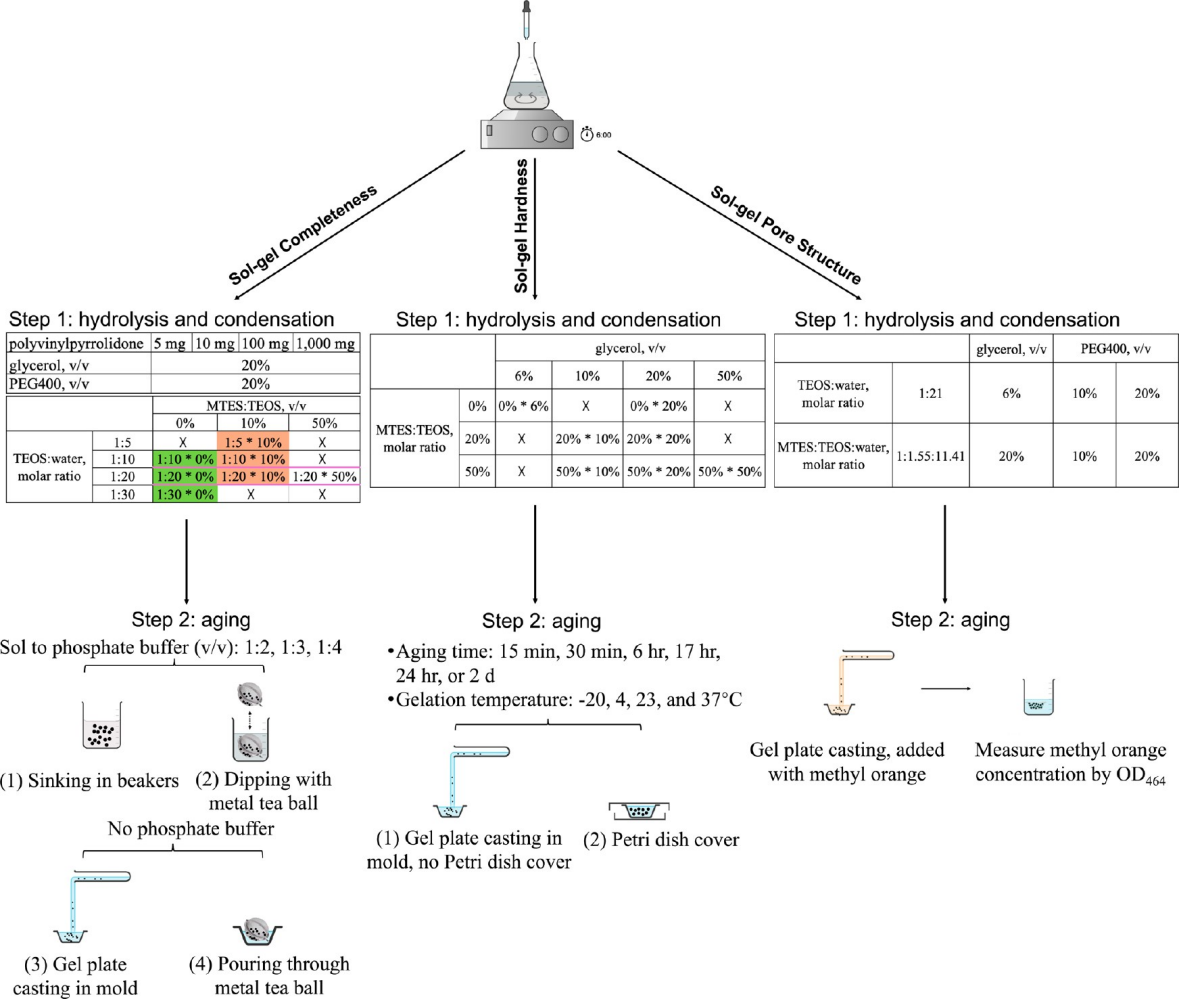
Summary of encapsulation procedures used to control sol–gel completeness, hardness, and pore structure.

To achieve the desired encapsulation, multiple parameters were systematically tested for their impacts on the sol–gel completeness, hardness, and pore structure (Figure 1). For complete coating, we evaluated a series of factors, including phosphate buffer, hydrolysis ratio, coating strategies, MTES addition, PVP addition, and glycerol/PEG400 addition. For gel hardness, we compared the impacts of aging temperature, phosphate buffer, MTES and glycerol addition, aging time, and dish cover. Lastly, the effect of adding different quantities of glycerol and PEG400 was investigated for controlling pore structure. We predicted that MTES addition, PVP addition, lower hydrolysis ratio, and a double coating would result in a more cohesive gel surface; that more MTES, longer aging time, and dish cover would yield a harder gel; and that more glycerol or PEG400 would increase pore size.

### Formulation of Various Precursor-Derived Gels

#### Tetraethyl Orthosilicate

TEOS was mixed with water in molar hydrolysis ratio of 20:1 (water to TEOS), and an acid catalyst was applied by adding 1 mol/L HCl to sol solution until pH = 1 to increase the hydrolysis rate of alkoxysilanes.^31^ The solution was continuously mixed on the platform shaker in the fume hood with open head space to allow complete hydrolysis and alcohol evaporation (at least 4 h). A strong base, 1 mol/L KOH, was added to adjust the sol solution to neutral pH. BC in phosphate buffer (35 mM; pH 8.0) was added into neutral sol solution in a ratio of 1:3 (v/v, sol solution: BC in phosphate buffer). Gelation occurred in 5 min at room temperature, and the final pH was about 8. This entire process is referred to as the “TEOS original recipe” below.

#### Others

Recipes of other precursors (e.g., sodium silicate) can be found in SI 1.1.

### Assessment of Encapsulation Recipe Variation Impacts on Sol–Gel Completeness

#### Gel Imaging

The sol–gel product made with each recipe adjustment was visualized by Scanning Electron Microscopy (SEM) using a Hitachi S-4800 (Tokyo, Japan). SEM images aimed to display coating completeness and pore structure (pore size and porosity) of sol–gel coated BCs. Various magnifications of SEM images (scale bars included in images) captured different portions of sol–gel coated BCs, providing both overall and detailed information on sol–gel coating.

#### Phosphate Buffer to Sol Solution Ratio

Phosphate buffer was employed to help stabilize pH of neutralized sol solution (buffer between pH 5.8 and 8.0) and avoid damaging cell viability during gelation. Phosphate buffer was chosen because it has been widely used in encapsulating bacterial suspensions with sol–gel and has less potential influences on enzyme activity compared to organic buffer (e.g., Tris).^21,44−47^ Thus, various ratios of sol solution to 35 mM phosphate buffer containing BC were compared to evaluate the impacts on the coating completeness. Nevertheless, the sol solution water content could increase the gelation time and potentially interfere with the coating performance; therefore, BC without phosphate buffer was also compared in treatment groups. Ratios of sol solution to phosphate buffer varied from no phosphate buffer to 1:2, 1:3, and 1:4 (v/v). Other steps were the same as those in the TEOS original recipe.

#### Coating Strategies

A variety of coating strategies were applied to encapsulate BCs with neutral sol solution to make coating more complete: (1) *Sinking in beakers (single coating vs double coating)*. A phosphate buffer with BC was poured into a beaker with neutralized sol solution, then was aged for 1 day. BCs were coated twice by repeating the same process. (2) *Dipping with metal tea ball (single coating vs double coating)*. Sol solution was first neutralized in a beaker with phosphate buffer, and then a metal tea ball holding BC was dipped in the neutral sol solution and dried for 2–5 min. The dip–dry–dip coating was repeated until the sol solution in the beaker became gelated, and then the coated BCs were aged in the fume hood for a day. This entire process was repeated one additional time following aging. (3) *Gel plate casting in mold*. BC without phosphate buffer was directly added to neutralized sol solution, fully vortexed for a minute, and poured to mold for casting and aging. Waxed paper with cylinder-shaped mold and hydrophobic plastic weighing boats were used as molds because both have nonadhesive surfaces that allow easy removal of the sol–gel product. The weighing boat is made from polystyrene, which is biologically inert and chemically resistant to diluted acids and bases. The resulting product was BCs randomly distributed within the regular shape of a gel plate. (4) *Pouring through metal tea ball*. BCs without a phosphate buffer were added to neutralized sol solution, fully vortexed for a minute, and then immediately poured into the tea ball. The filtrate sol solution was repeatedly poured onto the BC surface until gelation. The resulting product was individual BCs coated separately with the tea ball filtering redundant sol solution.

#### Hydrolysis Ratio

Before condensation, guaranteeing complete hydrolysis requires a hydrolysis ratio of water over silicon alkoxide to be >4.^48^ To evaluate the impacts of the hydrolysis ratio on encapsulation completeness, TEOS was mixed with water in a series of incremental hydrolysis ratios (water:TEOS) to make a sol solution: 10:1, 20:1, and 30:1. Other steps were the same as in the TEOS original recipe.

#### MTES Addition

Adding MTES could control the growth of silica particle size and thus improve sol–gel surface cohesiveness.^36^ To evaluate the impacts of MTES addition on sol–gel coating completeness, a sol solution was mixed at a hydrolysis ratio of 20:1 (water to TEOS) in addition to two MTES volumes: 10% and 50% (MTES:TOES, v/v). Different hydrolysis ratios of water to TEOS (10:1 and 5:1) were also added to 10% (MTES:TOES, v/v) MTES to check the coating completeness. Other steps were the same as in the TEOS original recipe.

#### Polyvinylpyrrolidone Addition

To evaluate whether adding PVP aided sol–gel coating completeness and cohesive smooth surface formation, different masses of PVP were added to sol solution prior to shaking for hydrolysis and ethanol evaporation: 1 g, 100 mg, 10 mg, or 5 mg. PVP was first dissolved in 12 mL of DI water and then added to either 20:1 (water:TEOS) or 0.5:0.5:20 (MTES:TEOS:water). BCs without phosphate buffer were directly added to neutralized sol solution containing 20% glycerol (v/v), fully vortexed for 1 min, and poured into the tea ball immediately. The other steps were the same as those in the TEOS original recipe.

#### Glycerol and PEG400 Addition

To test the impacts of glycerol and PEG400 on gel coating completeness, 20% glycerol or PEG400 was added to the sol solution (v/v), followed by pouring neutralized sol solution containing BC (no phosphate buffer) through a tea ball. Sol solution was mixed by either TEOS:water = 1:20 or MTES:TEOS:water = 0.5:0.5:20, together with 5 or 10 mg of PVP added. The coating process was conducted twice; other steps were the same as those in the TEOS original recipe.

### Assessment of Encapsulation Recipe Variation Impacts on Sol–Gel (Gel Plate) Hardness

The hardness of the gel plate was evaluated by controlling gelation temperature (−20, 4, 23, and 37 °C), MTES addition (20% or 50% molar of TEOS), glycerol addition (6%, 10%, 20%, or 50% of sol solution, v/v), aging time (15 min, 30 min, 6 h, 17 h, 24 h, or 2 d), Petri dish cover (covered or not covered), and hydrolysis ratios (1:20 or 1:4, TEOS:water, v/v). Petri dish cover was defined such that the gel plate was placed inside a Petri dish for aging while the cover was closed (or not closed) to compare the impacts of environment moisture during aging on gel plate hardness. Hardness of the gel plate was qualitatively evaluated by observational methods. Relative hardness was compared by applying consistent pressure onto the surface of the gel plate products and visually inspecting the resulting deformations (e.g., cracks on the gel plate surface, Figure S8). Additionally, the hardness was also tested by placing gel plates in K1 medium and applying continuous shaking at 150 rpm to observe the gel plate resistance to wear. The visual inspection approaches, albeit nonquantitative, were important for assessing the practical durability of the gel plates.

### Sol–Gel Porosity and Pore Size Adjustment by Glycerol and PEG400

As described above, glycerol or PEG400 was added to the neutralized sol together with MTES. Aside from checking impacts on sol–gel observational hardness and completeness, the addition of glycerol or PEG400 was also intended to control the sol–gel’s pore size and porosity by decreasing surface tension and osmotic stress on microbes. A desirable pore size should be larger than the target contaminant molecule size and smaller than the typical bacterial cell size. Thus, different portions of glycerol or PEG400 were added to the neutralized sol to compare with no glycerol or PEG400 addition. 6% glycerol, 10% PEG400, and 20% PEG400 (glycerol/PEG400:sol, v/v) were added in a sol solution of 21:1 (water:TEOS) hydrolysis ratio; 20% glycerol, 10% PEG400, and 20% PEG400 (glycerol/PEG400:sol, v/v) were added in a sol solution of 1:1.55:11.41 (MTES:TEOS:water) hydrolysis ratio. To check pore size, methyl orange (0.76 g/L), with 6–8 nm molecular diameter,^49^ was added to neutralized sol solution in 0.5% of total sol solution volume. Aged sol–gel was added to DI water, and OD_464_ of the solution was measured with Varian Cary 50 Bio UV–visible Spectrophotometry over time.

### Encapsulation of Biofilm-Enriched BCs with Sol–Gel Using Developed Recipes (Complete, Porous, and Highly Mechanical Stable Coating)

Methods for growing *Paraburkholderia xenovorans* LB400 (LB400) biofilms on BCs can be found in our prior work.^16^ Briefly, LB400 cells were grown in K1 medium (250 mL) containing biphenyl crystal (0.19 g) until cell cultures reached midexponential phase (OD_600_ = 0.5–0.6), then LB400 concentrated via centrifugation (3 mL, OD_600_ = 1) were resuspended in K1 medium (27 mL) containing corn kernel biochar (0.9 g) and biphenyl crystal (22.8 mg) for 10-days of growth. LB400 biofilm-BC was encapsulated with the tea ball approach to form individually coated biofilm-BCs. Sol solution was first prepared by mixing at a hydrolysis ratio of 1:20 (TEOS:water) or 0.5:0.5:20 (MTES:TEOS:water), with 10 mg of PVP added, followed by shaking at 200 rpm in a fume hood for 6–7 h. Sol solution was then neutralized by 1 mol/L KOH and 20% autoclaved glycerol or PEG400 (v/v) in a 15 mL centrifuge tube, vortexed for 1 min, and then biofilm-BCs were added into the tube. The biofilm-BCs together with sol solution were fully vortexed for 10–15s and poured into the autoclaved tea ball immediately. Residual sol solution was collected using the weighing boat (made from polystyrene, wiped with 70% ethanol) and repeatedly poured onto biofilm-BCs until gelation occurred. Coated biofilm-BCs were aged for 1 day in a sterile beaker and then the entire coating processes were repeated for the double-coated biofilm-BCs.

The process of sol–gel coating LB400 biofilm-BCs also occurred in the gel plate. Sol solution was first mixed at the hydrolysis ratio of 21:1 (water:TEOS) or 1:1.55:11.41 (MTES:TEOS:water), by shaking in the fume hood at 200 rpm for 6–7 h. Each sol solution was neutralized with 1 M KOH and autoclaved glycerol (6% or 20%, v/v) or PEG400 (20%, v/v) in a 15 mL centrifuge tube and vortexed for 1 min before biofilm-BCs were added into the tube. The biofilm-BCs together with sol solution were fully vortexed for 10–15 s and then immediately poured onto the weighing boat (wiped with 70% ethanol). Coated biofilm-BCs were aged under a sterile Petri dish cover; gel plates made with TEOS-only sol solution were aged for <19 h while gel plates made with MTES addition were aged for <1 h.

### Evaluation of Coated-Biofilms Viability and Biodegradation Potential

To investigate the performance of sol–gel protecting biofilm viability and integrity, live and dead cell distributions of LB400 biofilms on BCs were compared between noncoated biofilms and coated biofilms made from different recipes. Coated and noncoated biofilms were treated in either three rounds of reuse (10 mM benzoate in each round; biofilms were transferred to fresh media for reuse) for 25 days or carbon source starving conditions (only one time 10 mM benzoate addition) for 3 months. The live/dead cell distribution before and after treatment was stained with SYTO9 and propidium iodide (PI) following the instruction of LIVE/DEAD *Bac*Light Bacterial Viability Kit (Invitrogen, Waltham, WA) with minor adjustment (details in S1.2). Cells with a compromised membrane that were dead or dying stained red with PI, and cells with an intact membrane stained green with SYTO9. The live/dead cell distribution was then observed by Confocal Laser Scanning Microscopy (CLSM) with a Leica SP8 STED Super Resolution Confocal (Leica Microsystems, Exton, PA). Details of CLSM methods can be found in our prior work.^16^

To evaluate the biodegradation potential of sol–gel coated biofilm-BCs, both individually coated biofilm-BCs and gel plate coated biofilm-BCs made with various recipes (details in Figure 6, S13 captions) were added to K1 medium containing benzoate (10 mM) or acetate (3 mM) to track biodegradation. Benzoate and acetate were chosen as model chemicals to test the activity of coated cells and biofilms because both are less sorptive, more soluble, and thus more easily measured than PCBs in the aqueous phase.^16^ Benzoate and acetate concentrations were quantified over time using high performance liquid chromatography (Agilent 1100 series) and Ion Chromatography (Dionex ICS-2100), respectively. Details of carbon source measurement can be found in S1.3 and our prior work.^16^

### Test of Environmental Condition Impacts on Individually Coated BCs

To evaluate the impacts of both salinity and shear force on sol–gel surface integrity, 0.06 g of individually coated BCs were added to 20 mL of either high salinity solution (30 g/L sea salt in DI water, pH = 7) or K1 medium for continuous shaking at 150 rpm for one month. Individually coated BCs were made with double coating at a hydrolysis ratio of 0.5:0.5:20 (MTES:TEOS:water) with the addition of 10 mg of PVP, followed by sol solution mixing with 20% (v/v) of glycerol or PEG400. Sol–gel surface was visualized for comparing before and after the one month test using SEM imaging. The Brunauer–Emmett–Teller (BET) surface area and pore size distribution were measured by using a Quantachrome Nova 4200e, and the data collected were analyzed by the NovaWin software.

### Statistical Analysis

Statistical analysis was conducted in GraphPad Prism 9 and Microsoft Excel. Replication of each experiment was described in the figure caption. Normality of experimental data was tested by Shapiro–Wilk and Kolmogorov–Smirnov tests and establishing normal and log-normal probability plots. A pairwise two-sided *t* test or one-way ANOVA was applied to test for differences between treatment means depending on the experimental design. Differences were considered significant at the 95% confidence level (alpha = 0.05).

## Results and Discussion

### Characterization of the Optimal Coating Recipe

**Figure 2:**
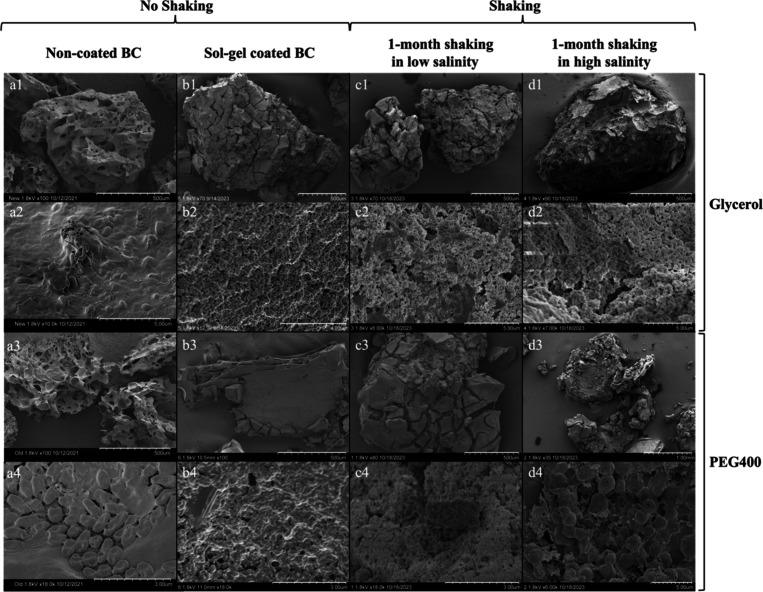
SEM imaging of sol–gel encapsulated-BC properties: completeness, observational hardness test, and porosity. No encapsulation: a1, a2, a3, a4; glycerol addition: b1, b2, c1, c2, d1, d2; PEG400 addition: b3, b4, c3, c4, d3, d4. Row “1” and “2” represented the samples made by the same recipe but visualized in different magnifications, and row “3” and “4” also represented the same sample in different magnifications. Each row has a similar magnification for comparison. Note the different magnifications in each image (see the scale bar).

Based on our encapsulation goals, we developed a recipe for coating BCs with desired sol–gel properties after iterative adjustment of the chemical compositions and coating strategies. Briefly, we mixed the sol solution MTES/TEOS/water/PVP (molar ratio = 0.5:0.5:20:7.7 × 10^–6^) at pH = 1 for 6–7 h, and vortexing 20% (v/v) of glycerol (MTDP-G) or PEG400 (MTDP-P), followed by KOH neutralization to pH = 7. BCs or biofilm-enriched BCs were added and filtered through a tea ball. Compared to noncoated BCs (Figure 2a), the BC surface was fully coated by sol–gel after aging (Figure 2b), and the sol–gel surface exhibited pore sizes (>4.6 nm radius for MTDP-G and 1.62 nm radius for MTDP-P) smaller than bacteria and larger than nutrient/pollutant molecules. In addition, the sol–gel structure was not damaged by shear force and high salinity after we shook encapsulated BCs in both low salinity (K1) and high salinity (30 g/L sea salt in DI water) for one month (Figure 2c and 2d). This indicates that both sol–gel recipes MTDP-G and MTDP-P yield encapsulated BCs with continuous, complete, porous, and high observational hardness surfaces.

### Impacts of Coating Parameters on Sol–Gel Completeness

#### Hydrolysis Ratio

Adjusting the hydrolysis ratio (water:TEOS) alone improved coating completeness, but a complete sol–gel coating did not develop on BC surfaces even though theoretical complete hydrolysis was achieved in the sol solution. Through SEM images of coated BCs (Figure S1 and 3a), we observed that the lower hydrolysis ratio (10:1) formed more complete coating and fewer cracks on sol–gel surfaces than the higher hydrolysis ratio (30:1); however, all three ratios yielded incomplete coatings. More BC surfaces were covered by gel made under lower hydrolysis ratios (10:1, 20:1), potentially because increasing hydrolysis ratio promotes siloxane bond cleavage reactions in the gel.^31^ Therefore, lower hydrolysis ratios were selected for later testing.

**Figure 3:**
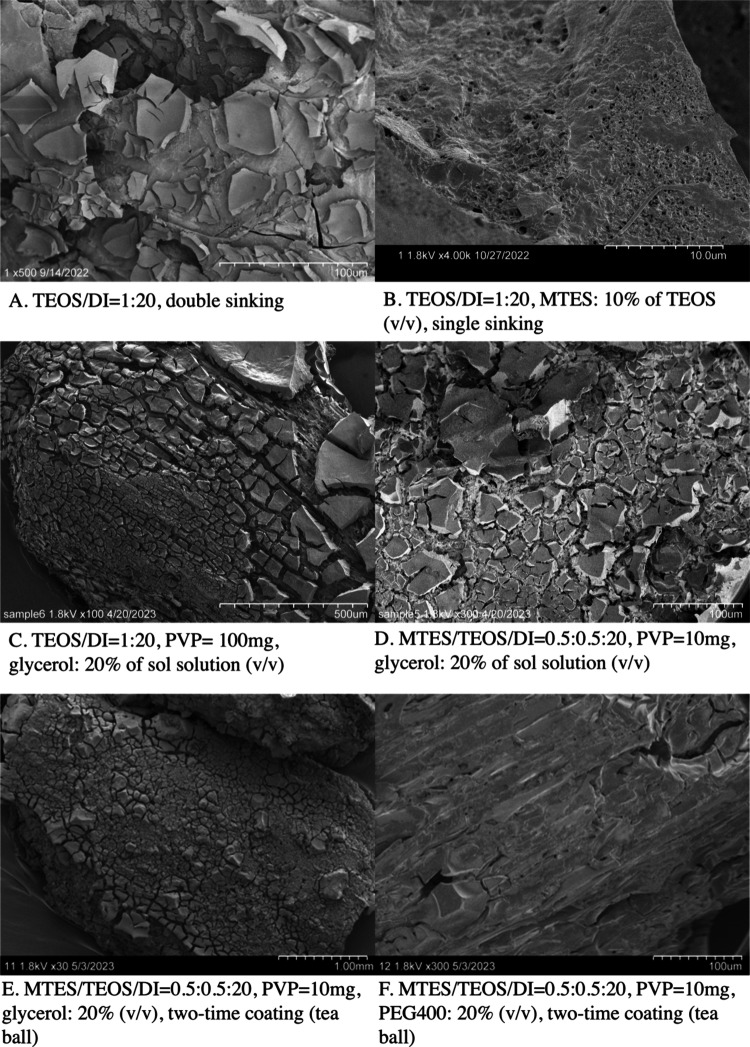
SEM images of sol–gel coated black carbons. Subpanels visually portray the effects of each factor on developing a complete, porous, and high observational hardness sol–gel encapsulation. All images were black carbon covered by a sol–gel made by different recipes. Recipe-A represents the factor of the hydrolysis ratio: TEOS/water = 1:20, double sinking; recipe-B represents the factor of MTES addition: TEOS/water = 1:20, MTES: 10% of TEOS (v/v), single sinking; recipe-C represents the factor of PVP addition: TEOS/water = 1:20, PVP = 100 mg, glycerol: 20% sol solution (v/v), single coating; recipe-D represents the factor of PVP addition: MTES/TEOS/water = 0.5:0.5:20, PVP = 10 mg, glycerol: 20% sol solution (v/v), single coating; recipe-E represents the factor of glycerol addition: MTES/TEOS/water = 0.5:0.5:20, PVP = 10 mg, glycerol: 20% (v/v), two-time coating (tea ball); recipe-F represents the factor of PEG400 addition: MTES/TEOS/water = 0.5:0.5:20, PVP = 10 mg, PEG400: 20% (v/v), two-time coating (tea ball). Recipe-E and F were the optimal sol–gel encapsulation recipes = MTES/TEOS/water = 0.5:0.5:20, PVP = 10 mg, glycerol/PEG400: 20% (v/v), two-time coating (tea ball).

#### Coating Strategies

To address the incomplete coating issues, we evaluated various coating strategies on the sol–gel surface coverage completeness. We compared the coating performance between sinking in beakers and dipping with tea ball approaches, as well as between single- and double-coating (Figure S2). We found that there were sol–gel surface cracks by using both sinking and dipping approaches. Dipping once or twice yielded no difference in coating completeness, which was potentially due to less sol–gel contact between BCs and sol solution, while double sinking resulted in more coated surfaces and smaller pore sizes (generally 5–7 μm, some ∼200 μm) compared to single sinking, although overall coating was incomplete.

#### MTES Addition

Addition of MTES potentially inhibits SiO_2_ growth and thus mitigates sol–gel cracking problems, although there were many possible causes of the sol–gel surface cracks. When the size of silica particles becomes large, the gel can reportedly crack and spall.^36^ Thus, we added MTES in an effort to control particle size growth and concurrently adjust sol solution hydrophobicity, which could also affect pore size.^36^ Another way to avoid large silica particles is to control the current sol solution reaction time to between 4 and 24 h; here we found that when using MTES, the sol solution shaking time should be <7 h before the gel solidified. MTES addition to the sol solution improved the BC surface coverage by sol–gel. Some small BC pieces had the desired smooth sol–gel-coated surface, high porosity, and small pore size (Figures 3b). Nevertheless, a higher percentage of MTES addition (50%) or lower hydrolysis ratio (1:5) exhibited little difference regarding completeness and evenness compared to adding a lower percentage of MTES (10%) or higher hydrolysis ratio (1:10)(Figure S3b–3e).

#### PVP Addition

PVP was added in the sol solution to improve the film-forming thickness and cohesive sol–gel coating. PVP is reportedly useful in decreasing the tendency to crack, improving adhesion over the hydrophobic core, and manipulating pore size in the porous film.^40−42^ Multiple masses of PVP addition to the sol solution were tested: 100, 10, and 5 mg. We found that the PVP impacts on gel completeness were related to sol solution hydrophobicity. When the sol solution was more hydrophobic (e.g., sol solution + MTES), less PVP (10 mg) was superior in terms of coating completeness and evenness compared to 100 mg of PVP (Figures 3d and S4). When the sol solution was more hydrophilic (e.g., sol solution with only TEOS), higher PVP addition (100 mg) yielded a complete gel structure (Figure 3c), while most BC surfaces remained uncovered with 5–10 mg of PVP addition (Figure S4). Compared with no PVP addition, the sol–gel surface with PVP addition formed a thicker film and more coating regardless of coating strategies, although PVP in our study did not resolve gel surface cracks.

#### Glycerol and PEG Addition

Glycerol and PEG enhanced the sol–gel coating completeness and adjusted the gel pore size. Both glycerol and PEG were used to increase pore size by reducing surface tension thus decreasing capillary forces during gelation and increasing biocompatibility by reducing osmotic stress.^20^ Based on the results of gel plate pore size adjustments, the details of which are presented later, 20% (v/v) of glycerol or PEG400 was added to test their impacts on sol–gel pore size and coating performance, together with effects of single- or double-coating. SEM imaging shows that sol–gel (+PEG400) had little to no gel surface cracks and mitigated gel surface peel-off compared with sol–gel (+glycerol) (Figures 3e, 3f). With the addition of glycerol or PEG400, sol–gel with MTES addition (right four images in Figure S5) generated a more complete coating than only TEOS (left four images in Figure S5), and double coating (bottom four in Figure S5) improved coating completeness, cracks, and evenness of the MTES addition gel surface compared to single coating (upper four in Figure S5) but did not benefit the only TEOS recipe. A summary of the impacts of all coating parameters on sol–gel characteristics of an individual coating is listed in Table 1.

**Table 1:** Summary of Each Recipe Change Effect on Gel Surface of Individual Gel Coating Outcome

Factor				Impacts on Sol–Gel Characteristics				
Recipe Changes	Sol Solution	Experiments		Complete Coating	Crack	Porous Matrix	Mechanical Strength	Film Thickness
Hydrolysis ratio (molar ratio)	Water:TEOS =	10:1		10:1 > 30:1	10:1 < 30:1	No Change	No Change	No Change
		20:1						
		30:1						
Coating strategies	TEOS:Water = 1:20	single sink		Double sink > single sink	sink has cracks	Pore size: double sink < single sink	No Change	No Change
		double sink						
		single dip		double dip ≈ single dip	dip has cracks	No Change	No Change	No Change
		double dip						
MTES addition (MTES:TEOS, %, v/v)	TEOS:Water = 1:20	50% MTES		MTES: 10% ≫ 50%	MTES addition has cracks	Porosity and pore size increase with MTES increases	No Change	No Change
		10% MTES						
	TEOS:Water = 1:10	10% MTES		1:10 ≈ 1:5				
	TEOS:Water = 1:5							
PVP addition	TEOS:Water = 1:20 or MTES:TEOS:Water = 0.5:0.5:20	Glycerol = 20% of sol solution (v/v)	no PVP	PVP > no PVP	PVP addition has cracks	No Change	No Change	Increases with PVP increases
			5 mg	TEOS: 100 mg >10 mg >5 mg				
			10 mg	MTES: 10 mg >100 mg	TEOS: 100 mg >10 mg >5 mg			
			100 mg					
Glycerol and PEG400 addition (% of sol solution, v/v)	TEOS:Water = 1:20 or MTES:TEOS:Water = 0.5:0.5:20	PVP = 5 mg or 10 mg	20% glycerol, single coating	MTES > TEOS	Glycerol addition has cracks	Pore size: 1.62 nm radius for PEG400 and >4.6 nm radius for glycerol	No Change	No Change
			20% glycerol, double coating	PEG400 > glycerol				
			20% PEG400, single coating	MTES: double > single	PEG400 addition has no cracks			
			20% PEG400, double coating	TEOS: double ≈ single				

In summary, the sol solution recipe with MTES and 10 mg of PVP (MTES/TEOS/water/PVP = 0.5:0.5:20:7.7 × 10^–6^), together with 20% (v/v) PEG400 and double coating, yielded the coating performance with the least cracks and the most smooth, even, and complete sol–gel surfaces. Using the same coating procedures with 20% glycerol also yielded a desirable overall sol–gel structure, although there were surface cracks. Both PEG400 and glycerol addition resulted in pore sizes (1.62 nm radius for PEG400 and >4.6 nm radius for glycerol) larger than the target pollutant molecule and smaller than LB400 cells.

#### Phosphate Buffer to Sol Solution Ratio

Phosphate buffer addition did not improve the sol–gel coating completeness. Phosphate buffer, which can help maintain pH values between 5.8 and 8.0, may aid stability of the neutralized sol solution and thereby protect cell viability during gelation. Thus, we tested the impacts of phosphate buffer to sol solution volume ratios (2:1, 3:1, 4:1, v/v) on cell/biofilms and aging time (minutes to days) on gel status (wet to fully dried) and coating completeness (cell leaching)(Table S1). We discovered that a higher cell to sol ratio required a longer aging time to completely dry gels because more water needed to evaporate (Table S1). Nevertheless, we observed cell leaching and growth in the liquid solution containing carbon sources (acetate and benzoate), which indicated potentially incomplete cell coating when adding phosphate buffer in recipe. Additionally, phosphate buffer potentially interacted with the sol solution by competing for water molecules with sol–gel polymer chains because of the strong hydration ability of the ions and weakened gel structure leading to low mechanical strength.^50−52^

#### Coating Completeness of Gel Plate

To address the issues of coating completeness and cracking, we also tried to encapsulate BCs with a gel plate approach. Making a gel plate requires a casting or molding process; thus, we evaluated multiple casting variables including mold selection and phosphate buffer. A plastic weighing boat proved a superior mold compared to wax paper because the weighing boat can form a regular shape without damage (Figure S6). No phosphate buffer addition in sol solution could improve coating completeness and the observed hardness of the sol–gel compared with phosphate buffer addition. Specifically, the gel made with phosphate buffer hardened slowly and did not adhere well to BCs, while the no phosphate buffer gel decreased the gelation time from hours to minutes and could completely coat BCs. The ideal optimized product (Figure S6, Panel 4) was permeable with propidium iodide, indicating that chemical transfer within sol–gel occurs (Figure S7). In general, this feasible gel plate development method completely encapsulates all BCs within the sol–gel and solves cracking concerns by adding BCs to neutralized sol solution without a phosphate buffer, followed by gelation in a plastic weighing boat at room temperature or higher.

#### Impacts on Gel Plate Hardness

We tested the effects of wide ranges of temperature (−20, 4, 23, 37 °C) on gel formation during gelation and found that higher temperature promotes formation of higher observational hardness gels. Thus, we chose 23 °C for the remaining experiments based on convenience and relatively high gel hardness. Although the gel plate product (Figure S6, Panel 4) did not dissolve in either DI water or high salinity (40 g/L) solution for at least 2 weeks, coated BC particles could escape from sol–gel plate after 2 days shaking at 150 rpm because the gel partially broke down due to intense shear force. This result was undesirable from an experimental perspective because such disintegration complicates determining if pollutant biodegradation was a result of encapsulated BCs or partially by escaped BCs; furthermore, disintegration under shear would also be undesirable for field deployment. Thus, the observed hardness of sol–gel was controlled by incorporating MTES to avoid potential BC escapes from the gel plate. We tested the impacts of varying MTES additions (20% and 50% molar ratio, MTES/TEOS) and glycerol (10%, 20%, and 50% glycerol/sol solution, v/v). When combining these two chemical adjustments, the observational hardness ranked as follows: 10% glycerol +20% MTES > 20% glycerol +50% MTES > 20% glycerol +20% MTES or 10% glycerol +50% MTES > 50% glycerol +50% MTES > no MTES. Although MTES addition improved observational hardness, MTES also promoted sol–gel surface cracks, which potentially exacerbates BC leakage issues. Increased cracking occurred from recipes in the following rank: 10% glycerol +20% MTES > 20% glycerol +20% MTES or 10% glycerol +50% MTES > 50% glycerol +50% MTES > 20% glycerol +50% MTES > no MTES (Figure S8). To summarize, the addition of 20% glycerol +50% MTES lessened cracking and improved observational hardness for the final gel plate coating.

Cracking during the fast drying process when mechanical stress exceeded the material ability to elastically respond has been reported;^53^ we found adding MTES led to higher hardness and yielded a less elastic gel with more cracking. To mitigate the cracking issue from MTES, a slower drying process and a high moisture environment during gelation may be helpful. We therefore used a Petri dish cover to slow sol–gel evaporation and control the aging time before transferring to solution. The optimal aging time under the Petri dish cover was 19 h (TEOS only recipe) and 15 min (MTES addition recipe). Both procedures completely encapsulated BCs and resulting gel plates exhibited high observational hardness with no observed biochar escape after 1 week of shaking.

#### Impacts on pore structure

**Figure 4:**
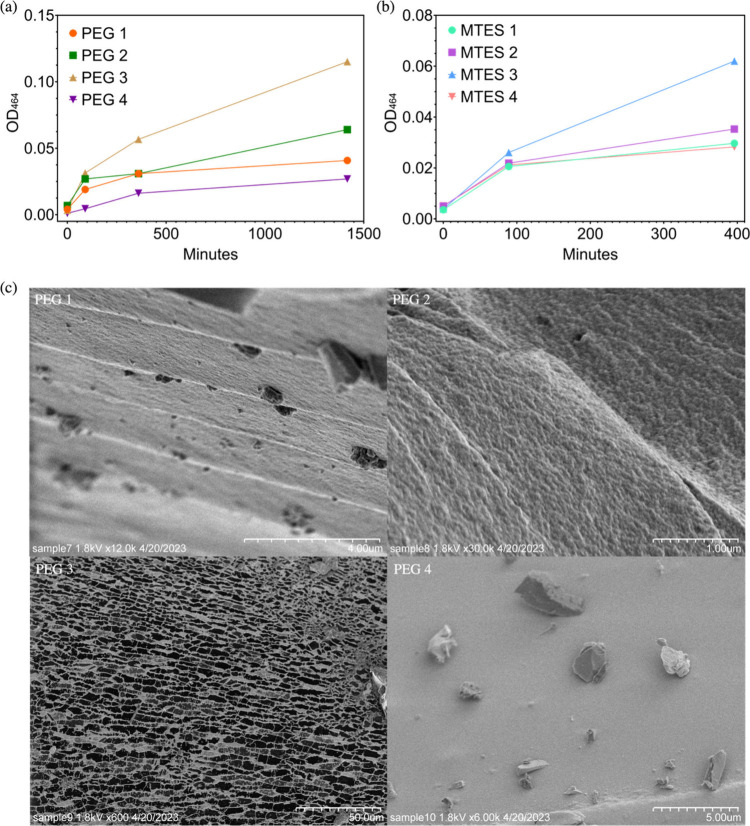
Methyl orange concentration was detected at OD_464_ through time with corresponding surface pore size (*n* = 1). (a) PEG1 represents 6% (v/v) glycerol addition; PEG2 represents 10% (v/v) PEG400 addition; PEG3 represents 20% (v/v) PEG400 addition; PEG4 represents no PEG400 or glycerol addition. (b) MTES 1: 20% (v/v) glycerol; MTES 2: 10% (v/v) PEG400; MTES 3: 20% (v/v) PEG400; MTES 4: no glycerol or PEG400. Gel in (a) was made with only TEOS (TEOS:water = 1:21) while gel in (b) had MTES addition (MTES:TEOS:water = 1:1.55:11.41). (c) SEM images of the sol–gel surface made by PEG 1–4 correspond to (a).

Although the gel plate coated BCs, produced by controlling aging time for both only TEOS and MTES addition recipes, could achieve complete BC surface coating and prevent BC escape after continuous shaking for 1 week, this improvement on encapsulation hampered benzoate biodegradation when testing encapsulated biofilm-enriched BCs. In contrast, incomplete encapsulation of biofilm-BCs, which could allow biofilm-BCs to escape the gel plate, did display benzoate biodegradation (Figure S9). We hypothesized that the no benzoate degradation phenomenon observed with encapsulated gel plates was due to gel pore size limiting pollutant mass transfer from the surrounding solution into coated biofilms. Diffusion rate of benzoate in sol–gel depends on the thickness and structure of sol–gel, and estimating the benzoate diffusion rate in sol–gel was beyond the scope of the current work. However, the diffusion rate would be an important parameter to standardize sol–gel product for future scale-up stages and incorporates quantitative SEM (e.g., energy dispersive X-ray spectroscopy) to control sol–gel thickness. Glycerol and PEG addition are reported to increase pore size and improve biocompatibility.^20^ We thus tested the diffusion capability of chemicals through existing pore size and the impacts that adding glycerol (0%, 6%, and 20%, v/v of sol solution) and PEG400 (0%, 10% and 20%, v/v of sol solution) in sol–gel recipe had on manipulating pore size. We added methyl orange in the sol solution prior to gelation on the premise that if the pore size of the gel increased, methyl orange would more easily diffuse and be detected at OD_464_ (methyl orange calibration curve in Figure S10). Based on this premise, the gel pore size was larger when either glycerol or PEG400 was added compared to no glycerol or PEG400 addition (Figure 4a and 4b). More specifically, a higher volume of PEG400 addition resulted in larger gel pore size, while glycerol did not increase the pore size of the gel recipe with MTES addition but did help with the only TEOS recipe. Similarly, the gel surface was rougher and more porous when PEG400 was added while no PEG400 or glycerol addition group resulted in a smooth surface with no pores (Figure 4c). Overall, the gel pore size with the tested glycerol and PEG400 addition ranked as follows: 20% PEG400 > 10% PEG400 > 6% glycerol > no glycerol or PEG400. A summary of the impacts of all coating parameters on gel plate characteristics are listed in Table 2.

**Table 2:** Summary of Each Recipe Change Effect on the Gel Surface of the Gel Plate Coating Outcome

Factor				Impacts on Sol–Gel Characteristics				
Recipe Changes	Sol Solution	Experiments		Complete Coating	Crack	Porous Matrix	Mechanical Strength	Film Thickness
Gelation temperature	TEOS:Water = 1:20	–20 °C		No Change	No Change	No Change	Increases with temperature increases	No Change
		4 °C						
		23 °C						
		37 °C						
Phosphate buffer (v/v, cell to sol solution)	TEOS:Water = 1:20	no phosphate buffer		No phosphate buffer addition > phosphate buffer addition	No Change	No Change	No phosphate buffer addition > phosphate buffer addition	No Change
		2:1						
		3:1						
		4:1						
MTES and glycerol addition (molar ratio, MTES/TEOS; glycerol/sol solution, v/v)	(TEOS+MTES):Water = 1:4	no MTES	10% glycerol	No Change	10% glycerol + 20% MTES > 20% glycerol + 20% MTES	No Change	10% glycerol +50% MTES > 50% glycerol + 50% MTES > no MTES	No Change
		20% MTES	20% glycerol		10% glycerol + 50% MTES > 50% glycerol + 50% MTES > 20% glycerol + 50% MTES > no MTES		10% glycerol + 20% MTES > 20% glycerol + 50% MTES > 20% glycerol + 20% MTES	
		50% MTES	50% glycerol					
Aging time and dish cover	TEOS:water:glycerol = 1:21:2.5	dish cover	<1 h	TEOS: 19 h works best		No Change	No Change	No Change
	MTES:TEOS:water:glycerol = 1:1.55:11.41:1.97	no dish cover	1–24 h	MTES+TOES: 15 min works best				
			>24 h					
Glycerol and PEG400 addition (v/v, % of sol solution)	TEOS:Water = 1:21	no glycerol or PEG400		No Change	No Change	20% PEG400 > 10% PEG400 > 6% glycerol > no glycerol or PEG400	No Change	No Change
		6% glycerol						
		10% PEG400						
		20% PEG400						
	MTES:TEOS:Water = 1:1.55:11.41	no glycerol or PEG400				20% PEG400 > 10% PEG400 > 20% glycerol > no glycerol or PEG400		
		20% glycerol						
		10% PEG400						
		20% PEG400						

### Cell Viability Enhancement: Individually Encapsulated Biofilm-BC Protects Cell Viability for at Least 3 Months without Carbon Source Supply

We compared the viability of biofilms on BC encapsulated with two different recipes before and after treatment with nonencapsulated biofilms by visualizing live and dead biomass on the BC surfaces. The two recipes evaluated were the optimal tea ball coating (recipes E and F in Figure 3), which formed the least cracks and was the most smooth, even, and complete sol–gel surface; and phosphate buffer addition (recipe-A in Figure 3), which maintained cell viability after coating as evidenced by acetate and benzoate biodegradation (chemical degradation data are presented below).

**Figure 5:**
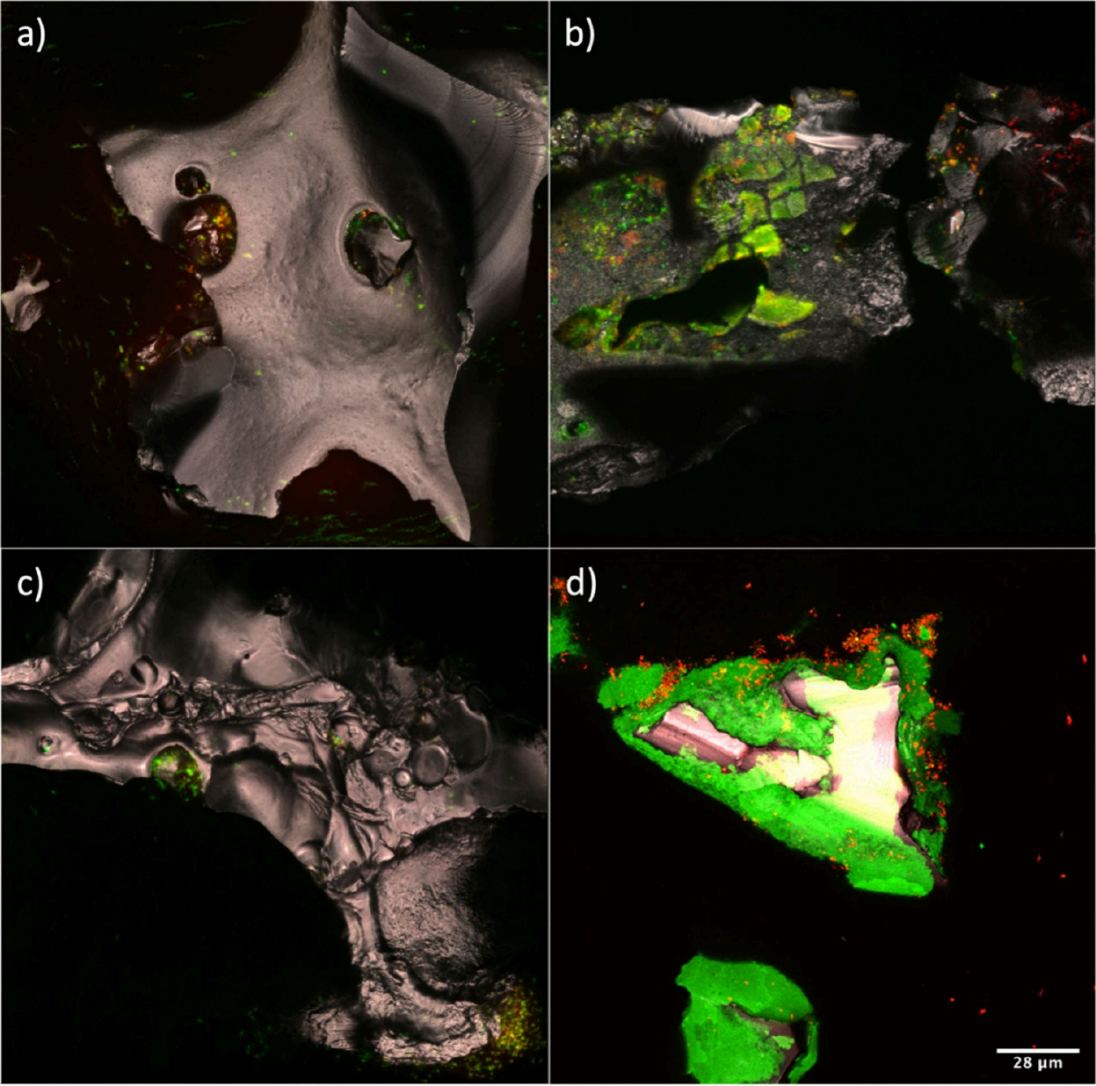
Cell viability of sol–gel encapsulated biofilm-BC. (a) Unencapsulated biofilm-BC after three rounds of reuse (10 mM benzoate in each round; biofilms were transferred to fresh media for reuse) within 25 days; (b) sol–gel coated biofilms by recipe-A before any treatment; (c) sol–gel coated biofilms by recipe-A after three rounds of reuse (10 mM benzoate in each round; biofilms were transferred to fresh media for reuse) within 25 days; (d) sol–gel coated biofilms by recipe-E after adding only one benzoate spike (10 mM) within three months. Recipe-A: 1:20 of TEOS:water, double sinking, 1:3 sol to phosphate buffer, aging under vacuum; recipe-E: glycerol (20%, v/v), MTES/TEOS/water = 0.5:0.5:20, PVP = 10 mg, double coating (tea ball)(MTDP-G). Green dots represent live LB400 cells, while red dots represent dead LB400 cells. Green continuous pieces in parts b and d result from sol–gel sorbing SYTO9 dye and emitting fluorescence.

Before treatment, we observed live LB400 cells (green dots) underneath the sol–gel made by recipe-A while the partial BC surface was covered by sol–gel (continuous green fluorescence, Figure 5b). Following treatment, both noncoated biofilms and biofilms coated by sol–gel recipe-A detached from BC surfaces with only limited amounts of LB400 cells remaining within the BC pore structure (Figure 5a and 5c). Importantly, sol–gel made by recipe-A also disappeared after treatment. This indicates that biofilm abundance, without sol–gel protection, decreased over time against continuous shear force, and sol–gel made by recipe-A only maintains gel integrity and protects biomass for less than 25 days. In contrast, after three months, sol–gel created by recipe-E&F still covered most BC surfaces as shown by continuous green fluorescence (Figure 5d and S11). Live cells (green dots) were noted inside the gel, although dead cells (red dots) were seen nearby the gel. This important finding indicates that sol–gel made with recipe-E&F not only prevented biomass from being washed away by continuous application of shear force, but also protected cell viability in the absence of carbon and energy sources for at least three months. In general, sol–gel made with recipe-E&F outperformed that with recipe-A in terms of complete biofilm-BC coating, maintaining sol–gel, and more importantly, biofilm integrity. Sol–gel (recipe-E&F) encapsulation maintained biofilm abundance for a longer period than nonencapsulated biofilms (90 days vs 25 days), which indicates the enhanced biodegradation potential in the presence of sol–gel.

### Chemical Degradation Proof-of-Concept: Individually Coated Biofilm-BC Exhibited Enhanced Biodegradation Potential over Gel Plate-Coated Biofilm

**Figure 6:**
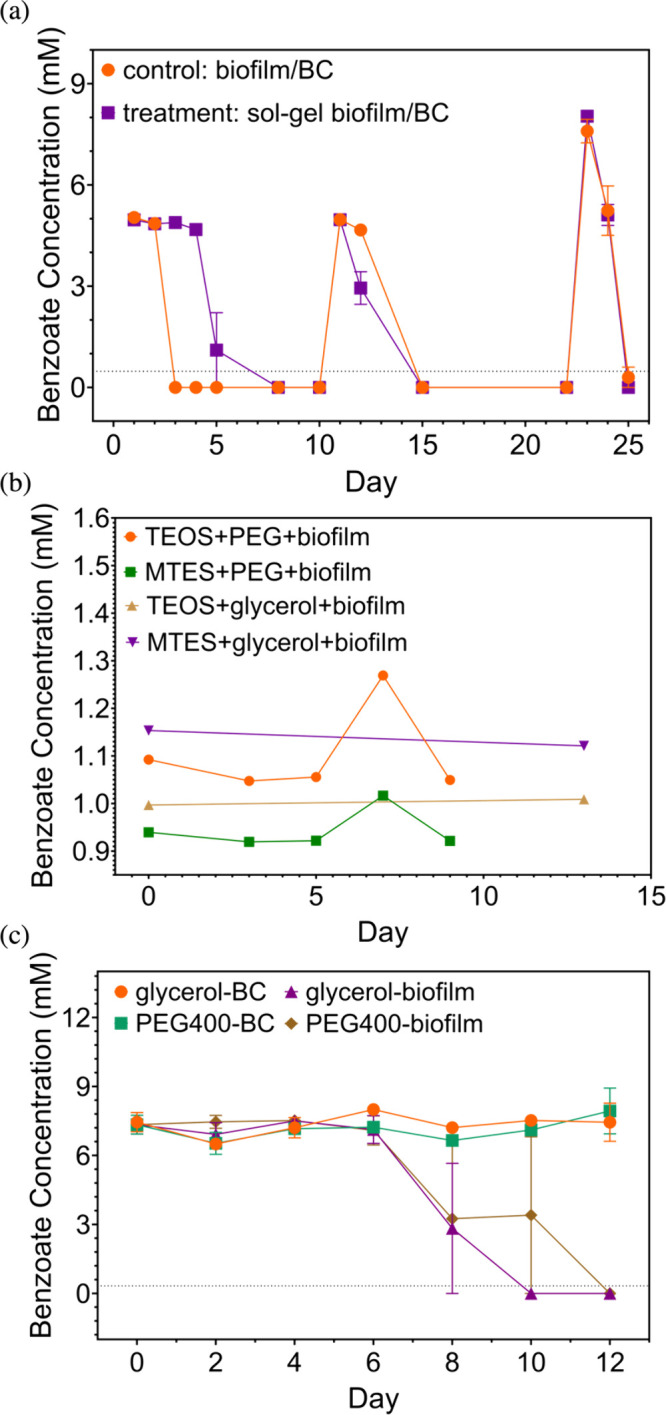
Benzoate biodegradation performance: (a) comparing benzoate degradation performance between encapsulated biofilm and nonencapsulated biofilm (*n* = 2), where biofilm was coated by recipe-A; error bars represent the standard error of biological duplicates; (b) comparing benzoate degradation between recipes with MTES addition and TEOS only (*n* = 1), where biofilm was coated by gel plate; (c) comparing benzoate degradation between encapsulated biofilms and encapsulated biochar (glycerol addition and PEG400 addition) (*n* = 2), where biofilm or biochar was coated by individual coating of adjusted recipe-E and recipe-F; error bars represent the standard error of biological duplicates. Dashed lines represent method detection levels. Recipe-A: 1:20 TEOS:water, double sinking, 1:3 sol to phosphate buffer, aging under vacuum. Gel plate recipe: 21:1 (water:TEOS) or 1:1.55:11.41 (MTES:TEOS:water), 20% (v/v) PEG400; 21:1 (water:TEOS) with 6% (v/v) glycerol or 1:1.55:11.41 (MTES:TEOS:water) with 20% (v/v) glycerol. Adjusted recipe-E: MTES/TEOS/water = 0.5:0.5:20, PVP = 10 mg with 20% (v/v) glycerol, single coating (tea ball); adjusted recipe-F: MTES/TEOS/water = 0.5:0.5:20, PVP = 10 mg with 20% (v/v) PEG400, single coating (tea ball). Note panel (b) was the negative result of gel plate coated biofilms, indicating that benzoate was not degraded by biofilms coated with a gel plate recipe.

To evaluate the biodegradation potential of coated biofilms, benzoate and acetate were chosen to compare carbon source degradation performance between sol–gel coated biofilms and noncoated biofilms. Acetate degradation is the first indication of the activity of coated biofilms, while benzoate is an inducer and product of PCB biodegradation pathway (*bph*) and is more easily measured in the aqueous phase than PCBs.^54^ Three sol–gel coating recipes were chosen: phosphate buffer addition (recipe-A), a gel plate that completely encapsulates biochar without escape (gel plate recipe), and individual coating by tea ball (adjusted recipe-E and recipe-F with single tea ball coating in Figure 3). Both noncoated biofilms and biofilms coated by recipe-A degraded benzoate well during three rounds of biofilm reuse (Figure 6a). Compared with noncoated biofilms, coated biofilms lagged for 2 days prior to consuming benzoate and acetate (Figure 6a (benzoate), Figure S12 (acetate)). Recipe-A coated biofilms degraded about 30% more benzoate than noncoated biofilms (*p* = 0.072, significant differences of remaining benzoate concentration for two groups at day-12) in the beginning of the second round of biofilm reuse, but the benzoate degradation differences were absent in the third round of biofilm recycle. This phenomenon may be related to insufficient biofilm protection by recipe-A sol–gel (Figure 6a and 6c), where biofilms and sol–gel detached from the BC surface within 25 days of treatment even with sol–gel encapsulation (Figure 5). Thus, for recipe-A, most of the biofilms, both noncoated and coated, were resuspended into the aqueous solution, and only partial biofilms were left on BC surface and brought over to fresh media in each round of reuse, resulting in the same degradation performance.

When different cell-to-sol solution ratios were applied to coat biofilms, both benzoate and acetate were readily degraded by coated biofilms and cells within 3 days, respectively (Figure S13). A higher ratio (4:1) degraded acetate significantly (*p* < 0.0019) faster than that of 2:1 or 3:1 (4:1: 0.048, 2:1 and 3:1: 0.0012 mM/h) in the first half degradation period, but cell leaching was also observed together with carbon source biodegradation.

Although we controlled the sol–gel pore size to allow chemical transfer for gel plate coated biofilms (Figure 6b), there was still no benzoate degradation by both the TEOS only gel and MTES addition gel after 9 and 13 days. One potential explanation for this phenomenon is that the high degree of sol–gel mass transfer resistance delayed benzoate diffusion to biofilms from the bulk liquid. The square-shaped gel plate did indeed prevent biomass from escaping; however, the encapsulation made biofilms contact with the chemicals difficult. In contrast, biofilms individually coated by adjusted recipe-E and F could completely degrade benzoate within 12 days (Figure 6c), where individual coating maintained the original granular structure of biofilm-BC and did not limit the direct contact between biofilms and chemicals. However, glycerol addition degraded benzoate marginally more efficiently (10 days) than PEG400 addition (12 days), potentially because of sol–gel porosity and pore size. Overall, although biofilms coated by recipe-A and adjusted recipe-E&F both degraded benzoate efficiently, recipe-A had severe biofilm escape issues and unstable gel integrity that may facilitate benzoate degradation, whereas the gel plate coated biofilms encountered degradation difficulty due to potential mass transfer limitations. To balance the trade-off between biodegradation efficiency and biofilm protection, adjusted recipe-E&F were optimal for our study purpose, but recipes can be tuned to match other study aims as well.

The results demonstrate that individual encapsulation in a matrix from TEOS and MTES precursors and incorporation of glycerol/PEG400 and PVP are effective in preserving biofilm viability and activity and transferring chemicals from the bulk liquid to the biofilm microenvironment on the BC surface. We suggest that sol–gel encapsulation protection of biofilm viability and activity could be related to the high hydrolysis ratio (TEOS and MTES) followed by raising pH that removed undesirable alcohol and improved cytocompatibility,^39^ that the addition of glycerol and PEG400 reduced osmotic stress on biofilms to protect cell integrity and increase sol–gel biocompatibility,^20,25^ and that sol–gel hardness, microstructure, and complete coverage on biofilms were precisely controlled by a combination of recipe adjustment to enable biofilm integrity.^31,39,40^ This work provides a platform for developing biofilm protection applications with different microbial biofilm types (i.e., aerobic and potentially anaerobic) growing on or attached to different solid surfaces. Moreover, given the high porosity and pore size of sol–gel, target pollutants with radius sizes varying from <75 nm to <27.5 nm can pass through the sol–gel depending on glycerol or PEG400 addition. These size ranges encompass a large array of chemicals, where PCBs have molecular size of at most ∼1.3 nm. Additionally, MTES content in sol solution increases the hydrophobicity of sol–gel and thus potentially affects the transfer of both hydrophobic (e.g., PCBs) and hydrophilic pollutants through gel via hydrophobic interactions;^55,56^ however, testing the impacts on hydrophobicity was outside the scope of this study. Target pollutants and BCs could therefore be accordingly adjusted/tuned based on the desired adsorption performance, where higher sorption tendency may benefit the contact between pollutants and biofilms, and has proven nontoxic to attached cells.^14,18^ Assessing the sorption capacity of sol–gel and biochar on specific target pollutants (e.g., PCBs) was beyond the scope of this study but will be important when evaluating pollutant biodegradation performance in future work. The porous structure of the sol–gel matrix also allows potential application in bioreactors where fluid with pollutants can pass over coated biofilms, and encapsulated biofilms could be reused given their high stability of encapsulant and maintained biomass and activity.

More importantly, this work demonstrates the potential capacity of the TEOS + MTES tuned sol–gel matrix for protecting biofilms that could be applied to *in situ* contaminated sediment remediation. The encapsulated biofilms can sustain and even prolong their biodegradation potential against adverse environmental conditions, such as limited carbon source supply and undesirable changes including high salinity and continuous shear force. Sol–gel can last for extended time periods in the environment because of its unique characteristics: chemically inert, mechanically strong, thermally stable, and resistant to microbial attack.^24,39,44,57^ TEOS-derived sol–gel has also proven durable in marine environments and has promising resistance to weathering and slurry erosion.^58,59^ The sol–gel matrix not only preserves high viable cell density given its high observational hardness and stability, but also possibly prevents biofilm maturation and detachment because the sol–gel restrains cell outgrowth.^20^ Overall, this novel development of sol–gel application on biofilms can enhance the longevity of cell activity and further extend the biodegradation time.

## Conclusions

This study is the first to develop a novel method for encapsulating PCB-degrading aerobic biofilm-enriched biochar with sol–gel. Prior studies have focused more on encapsulation of living cells in sol–gel matrix mainly for storage purposes,^21,25,33^ but to the best of our knowledge no study successfully immobilized biofilms growing on solid surface for remediation. The promising features of this sol–gel approach lie in its capability to protect coated biofilms against adverse environmental changes (i.e., shear force and high salinity) and maintain biofilm viability and activity via its porous, well-covered, and mechanically stable gel matrix. Particularly, these environments are normally unfavorable to microbial cells (e.g., suspended cells and noncoated biofilms).^19,60^ More interestingly, the proposed method allows coated biofilms to remain viable without carbon sources for over three months, which exhibits great potential to extend the biodegradation reaction time. This novel technology development provides important guidance for designing microbe/material combinations for bioremediation purposes.

This research also holds important implications for improving *in situ* sediment remediation approaches that mitigate exposure to a pressing environmental toxicant. LC-PCBs, as the most volatile PCBs, cause direct exposure of sediment to humans and ecosystems. This newly developed approach can potentially extend LC-PCB biodegradation activity by a protective sol–gel layer on biofilm-enriched biochar, and sol–gel encapsulated biofilms offer the advantages of high stability and long-time survival in harsh environments. Our novel approach holds the potential to improve bioremediation efforts that decrease the PCB mass load in sediment and mitigate the sediment to air exposure pathway, thereby benefiting public health and ecosystems.

## Supporting Information Available

Supporting experimental methods for gel formulation; live/dead cell staining; carbon source measurement; SEM images of sol–gel coated black carbons by adjusting hydrolysis ratios (Figure S1); SEM images of sol–gel coated black carbons by adjusting coating strategies (Figure S2); SEM images of sol–gel coated black carbons by adjusting MTES addition (Figure S3); black carbons coated by gel made in different PVP addition (Figure S4); SEM images of sol–gel coated black carbons by adjusting PEG400 and glycerol addition (Figure S5); the impacts of phosphate buffer to sol solution ratios and gel aging time on gel status of coated LB400 cells and LB400 biofilm-BC (Table S1); the product of gel plates using different casting molds (Figure S6); partial gel plate of No. 4 in Figure S6 was permeable with propidium iodide (Figure S7); gel plates with different MTES and glycerol additions (Figure S8); benzoate degradation over time by various gel plates (Figure S9); calibration curve of methyl orange concentration over OD_464_ (Figure S10); cell viability of sol–gel encapsulated biofilm-BC after adding only one benzoate spike (10 mM) within three months (Figure S11); acetate degradation of LB400 biofilms coated by various sol to phosphate buffer ratios over time (Figure S12); benzoate and acetate biodegradation of LB400 cells and biofilms coated by various sol to phosphate buffer ratios (Figure S13) (PDF)
