# Supplementary material for: Development of a Novel PCB-Degrading Biofilm Enriched Biochar Encapsulated with Sol–Gel: A Protective Layer to Sustain Biodegradation Activity
Source: ACS ES T Eng. 2025 Mar 6;5(4):883–98. doi: 10.1021/acsestengg.4c00718 (PMC11998003; doi:10.1021/acsestengg.4c00718)
Supplement: Supplementary file 1 — ee4c00718_si_001.pdf [file ee4c00718_si_001.pdf]

Supporting Information

Development of a Novel PCB-Degrading Biofilm Enriched Biochar Encapsulated with Sol-Gel: A Protective Layer to Sustain Biodegradation Activity

Qin Dong,[†,‡] *Timothy E. Mattes*[†,‡,*] and *Gregory H. LeFevre*[†,‡,*]

[†] Department of Civil and Environmental Engineering, University of Iowa, 4105 Seamans Center, Iowa City, Iowa, 52242, United States

[‡] IIHR—Hydroscience and Engineering, University of Iowa, 100 C. Maxwell Stanley Hydraulics Laboratory, Iowa City, Iowa, 52242, United States

[*]**Corresponding Authors**: TEM: tim-mattes@uiowa.edu; Phone: +319 335 5065, Department of Civil and Environmental Engineering, 4112 Seamans Center for Engineering, University of Iowa, Iowa City IA, 52242, United States. GHL: gregory-lefevre@uiowa.edu; Phone: +319 335 5655, Department of Civil and Environmental Engineering, 4106 Seamans Center for Engineering, University of Iowa, Iowa City IA, 52242, United States.

This supplemental information document includes:
Supplemental methods (Section S1.1-1.3), Supplemental results (Figures S1-S13, Table S1). 13 Total Page (inclusive of this page).

S1. Supplemental Methods and Materials

S1.1 Formulation of Sodium Silicate-Derived Gels

One gram of sodium silicate (Sodium Metasilicate Pentahydrate, Sigma-Aldrich) was mixed with 6 mL DI water (type II water) and incubated at 80 °C until colloidal suspension. The suspension was cooled to room temperature, and pH was adjusted to 6.5 with 0.75 M citric acid. BC solution (BC in DI water) was mixed with colloidal suspension in a 1:1 volume ratio. The solution was left for 2 minutes for gelation and aging overnight.

S1.2 Live/Dead Cell Staining

Three microliters of both SYTO9 and propidium iodide (PI) were mixed in 994 µL of phosphate buffer saline (PBS), vortexed for thoroughly mixing. Sol-gel coated or non-coated biofilm-enriched biochar (~0.1 g) was added in the dye mixture solution (1 mL). Pipette mixing was applied to fully mix dye solution and biofilm samples, and then incubate in the dark at room temperature for 30 minutes. Dye solution was removed with pipettes after staining, and stained samples were rinsed with 1 mL PBS for three times. Samples were covered in 90% glycerol with 10 mM Tris-HCl (pH 8) and ready for Confocal Laser Scanning Microscopic imaging.

S1.3 Carbon Source Measurement

Benzoate concentration was quantified over time using high performance liquid chromatography (HPLC, Agilent 1100 series) with Diode-Array Detection (DAD) or Variable Wavelength Detectors (VWDs) and separated on a Sprite Targa reverse-phase C18 column (40 mm ×2.1 mm ×5 µm) with a PEEK guard column. Acetate concentrations were measured by Ion Chromatography (Dionex ICS-2100) equipped with AS11-HC (250 ×4 mm) anion exchange column and an AG11 guard column.

S2. Supplemental Results and Discussion

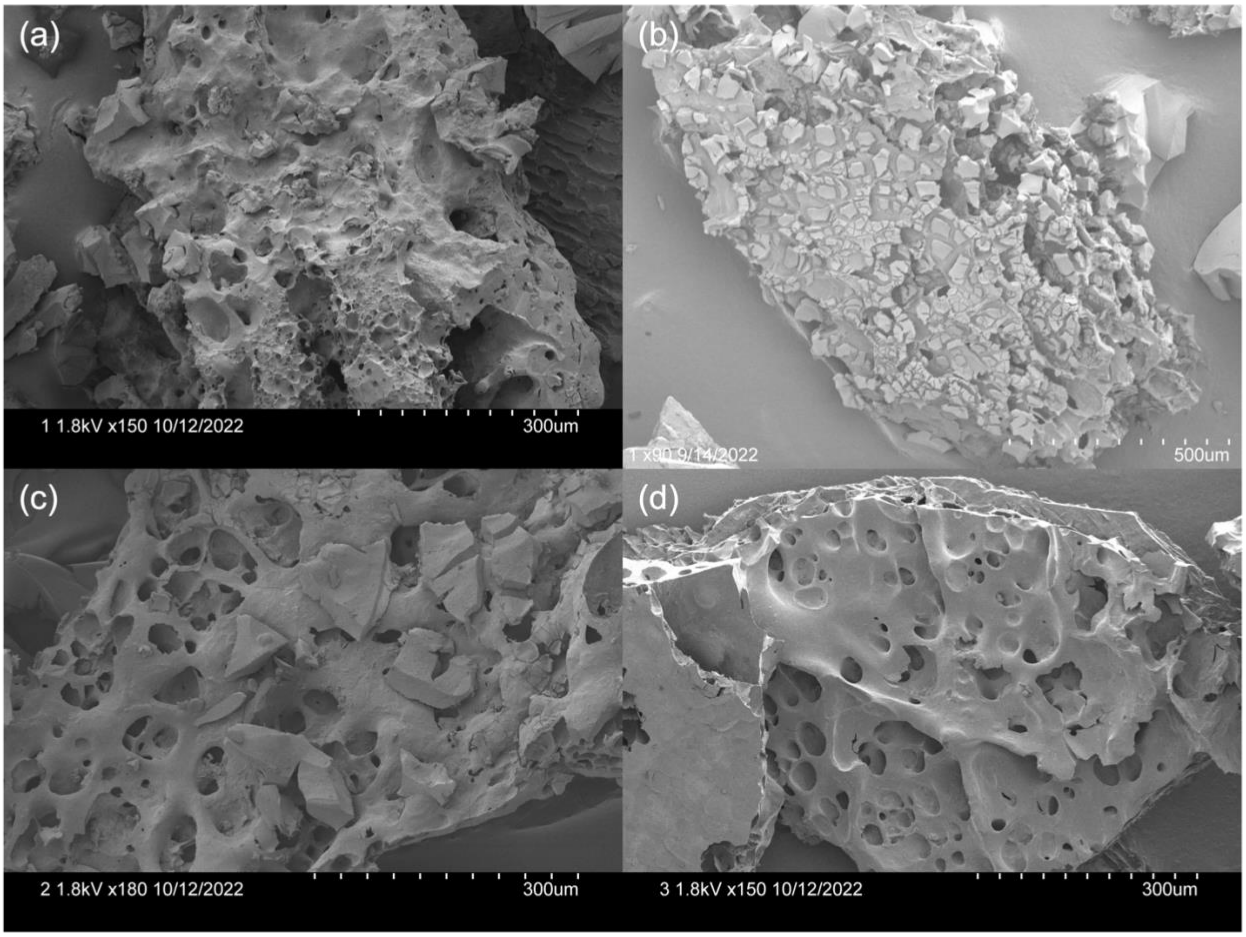

Figure S1. Scanning Electron Microscopy (SEM) images of sol-gel coated black carbons by adjusting hydrolysis ratios. (a) 10:1 (water:TEOS) hydrolysis ratio with 1:3 sol to phosphate buffer (double sinking); (b) 20:1 (water:TEOS) hydrolysis ratio with 1:3 sol to phosphate buffer (double sinking); (c) 30:1 (water:TEOS) with 1:3 sol to phosphate buffer (double sinking); (d) no coating.

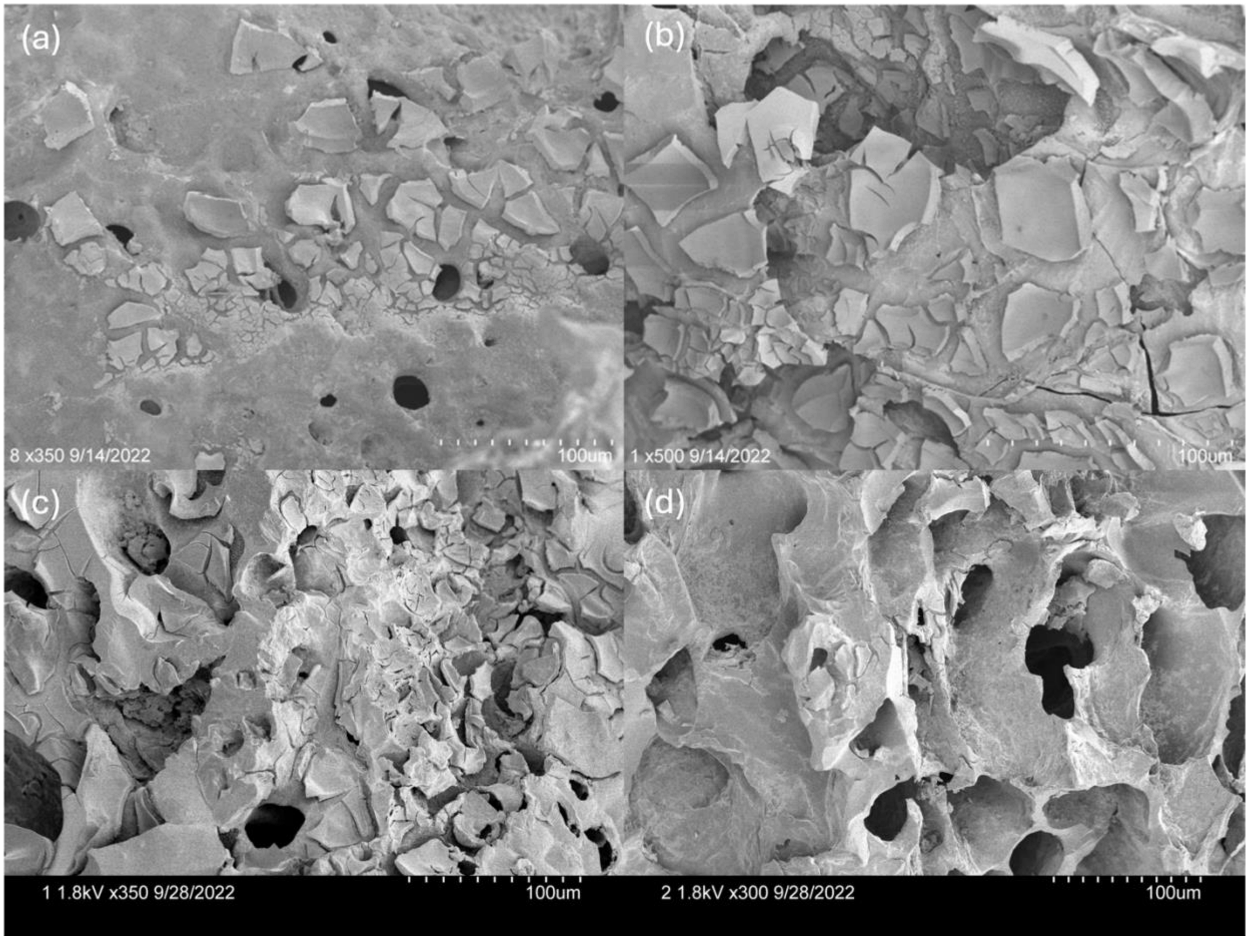

Figure S2. SEM images of sol-gel coated black carbons by adjusting coating strategies. (a) single coating of sinking in beakers; (b) double coating of sinking in beakers; (c) single dipping with tea ball; (d) double dipping with tea ball.

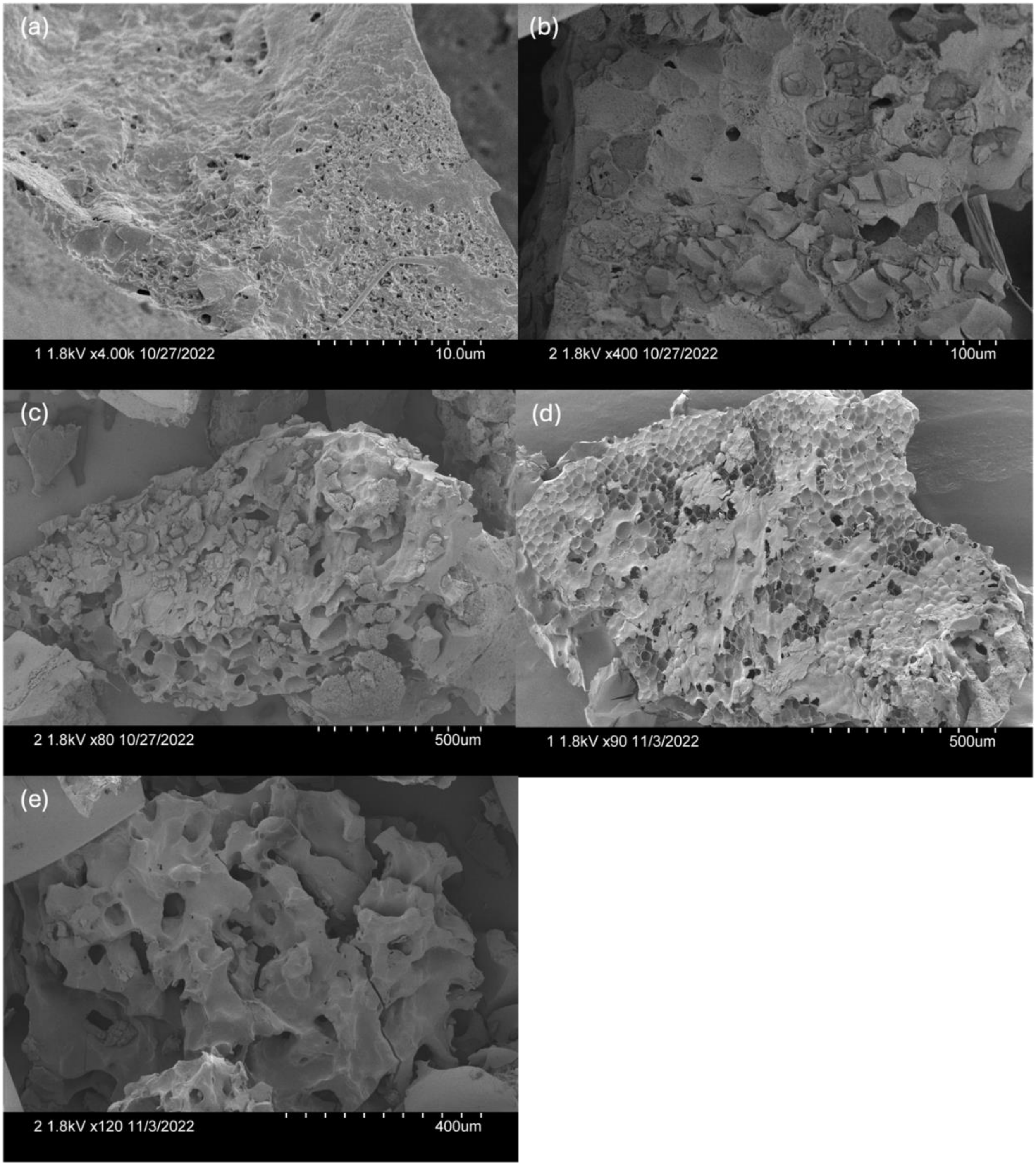

Figure S3. SEM images of sol-gel coated black carbons by adjusting MTES addition. (a) 10% MTES addition to 20:1 (water:TEOS) with single sinking; (b) 10% MTES addition to 20:1 (water:TEOS) with double sinking; (c) 50% MTES addition to 20:1 (water:TEOS) with single sinking; (d) 10% MTES addition to 10:1 (water:TEOS) with single sinking; (e) 10% MTES addition to 5:1 (water:TEOS) with single sinking.

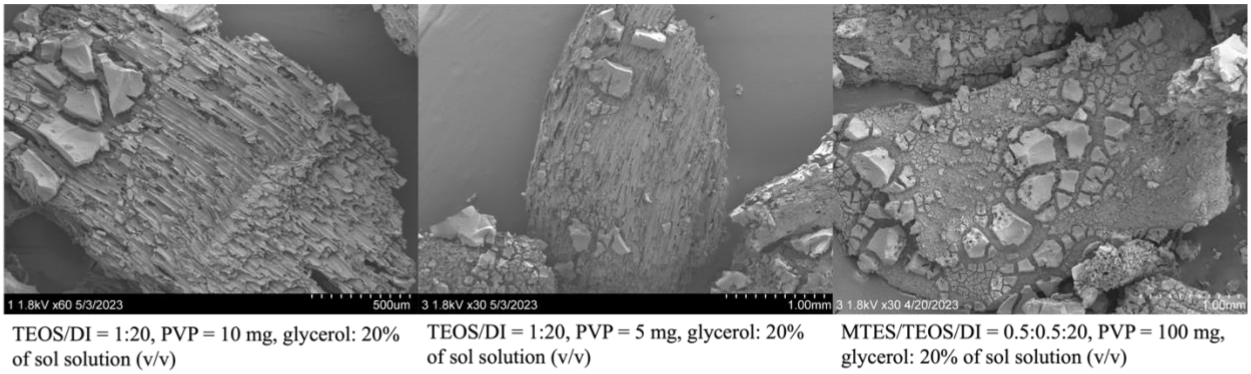

Figure S4. Black carbons coated by gel made in different polyvinylpyrrolidone (PVP) addition. Recipe in the left figure: TEOS/DI = 1:20, PVP = 10 mg, glycerol: 20% of sol solution (v/v). Recipe in the middle figure: TEOS/DI = 1:20, PVP = 5 mg, glycerol: 20% of sol solution (v/v). Recipe in the right figure: MTES/TEOS/DI = 0.5:0.5:20, PVP = 100 mg, glycerol: 20% of sol solution (v/v).

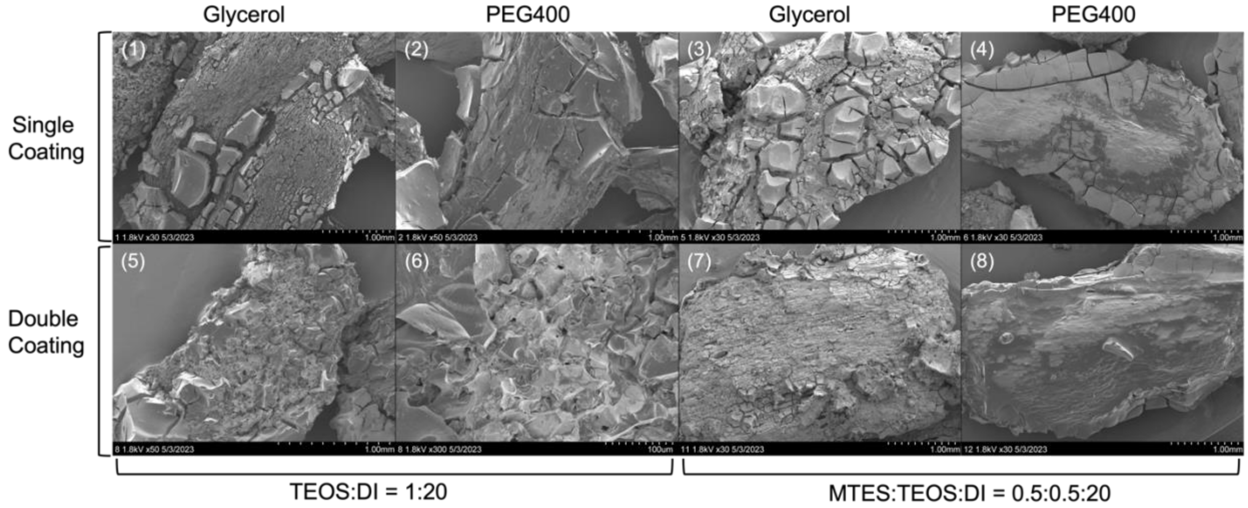

Figure S5. SEM images of sol-gel coated black carbons by adjusting PEG400 and glycerol addition. (1) TEOS/DI = 1:20, PVP = 10 mg, 20% (v/v) glycerol, one time coating; (2) TEOS/DI = 1:20, PVP = 10 mg, 20% (v/v) PEG400, one time coating; (3) MTES/TEOS/DI = 0.5:0.5:20, PVP = 10 mg, 20% (v/v) glycerol, one time coating; (4) MTES/TEOS/DI = 0.5:0.5:20, PVP = 10 mg, 20% (v/v) PEG400, one time coating; (5) TEOS/DI = 1:20, PVP = 10 mg, 20% (v/v) glycerol, two-time coating; (6) TEOS/DI = 1:20, PVP = 10 mg, 20% (v/v) PEG400, two-time coating; (7) MTES/TEOS/DI = 0.5:0.5:20, PVP = 10 mg, 20% (v/v) glycerol, two-time coating; (8) MTES/TEOS/DI = 0.5:0.5:20, PVP = 10 mg, 20% (v/v) PEG400, two-time coating.

Table S1. The impacts of phosphate buffer to sol solution ratios and gel aging time on gel status of coated LB400 cells and LB400 biofilm-BC. “1:2” referred to LB400 cell culture coating, while “1:3” and “1:4” referred to LB400 biofilm-BC coating.

Phosphate buffer to sol solution ratio	Aging time		
	Wet	Medium dried	Dried
1:2	<30 min	3 days	5 days
1:3	<30 min	N/A	6 days
1:4	<30 min	6 days	10 days

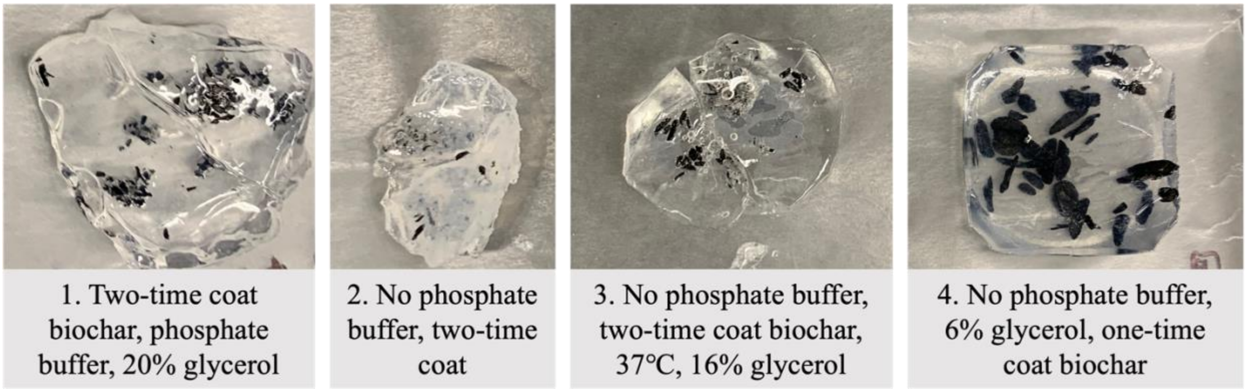

Figure S6. The product of gel plates using different casting molds. 1. Gel plate was made by encapsulating biochar in recipe of 1:20 (TEOS:water), double coating, 1:3 (sol solution:phosphate buffer, v/v), 20% (v/v) glycerol, aging at room temperature, and using waxed paper with a cylinder-shaped holder as mold. 2. Gel plate was made by encapsulating biochar in recipe of 1:20 (TEOS:water), double coating, no phosphate buffer, no glycerol, aging at room temperature, and using waxed paper with a cylinder-shaped holder as mold. 3. Gel plate was made by encapsulating biochar in recipe of 1:20 (TEOS:water), double coating, no phosphate buffer, 16% (v/v) glycerol, aging at 37 °C, and using waxed paper with a cylinder-shaped holder as mold. 4. Gel plate was made by encapsulating biochar in recipe of 1:20 (TEOS:water), single coating, no phosphate buffer, 6% (v/v) glycerol, aging at room temperature, and using a plastic weighing boat as mold.

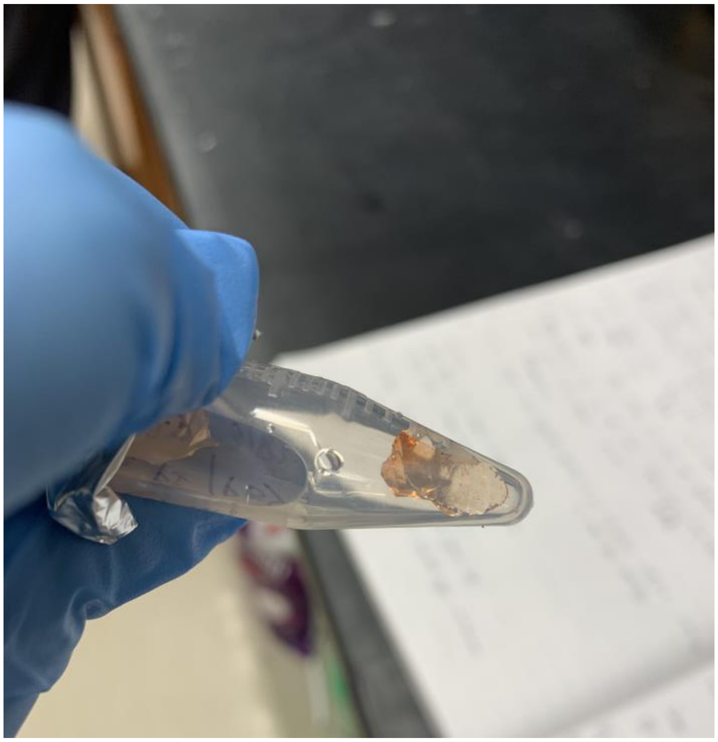

Figure S7. Partial gel plate of No.4 in Figure S6 was permeable with propidium iodide.

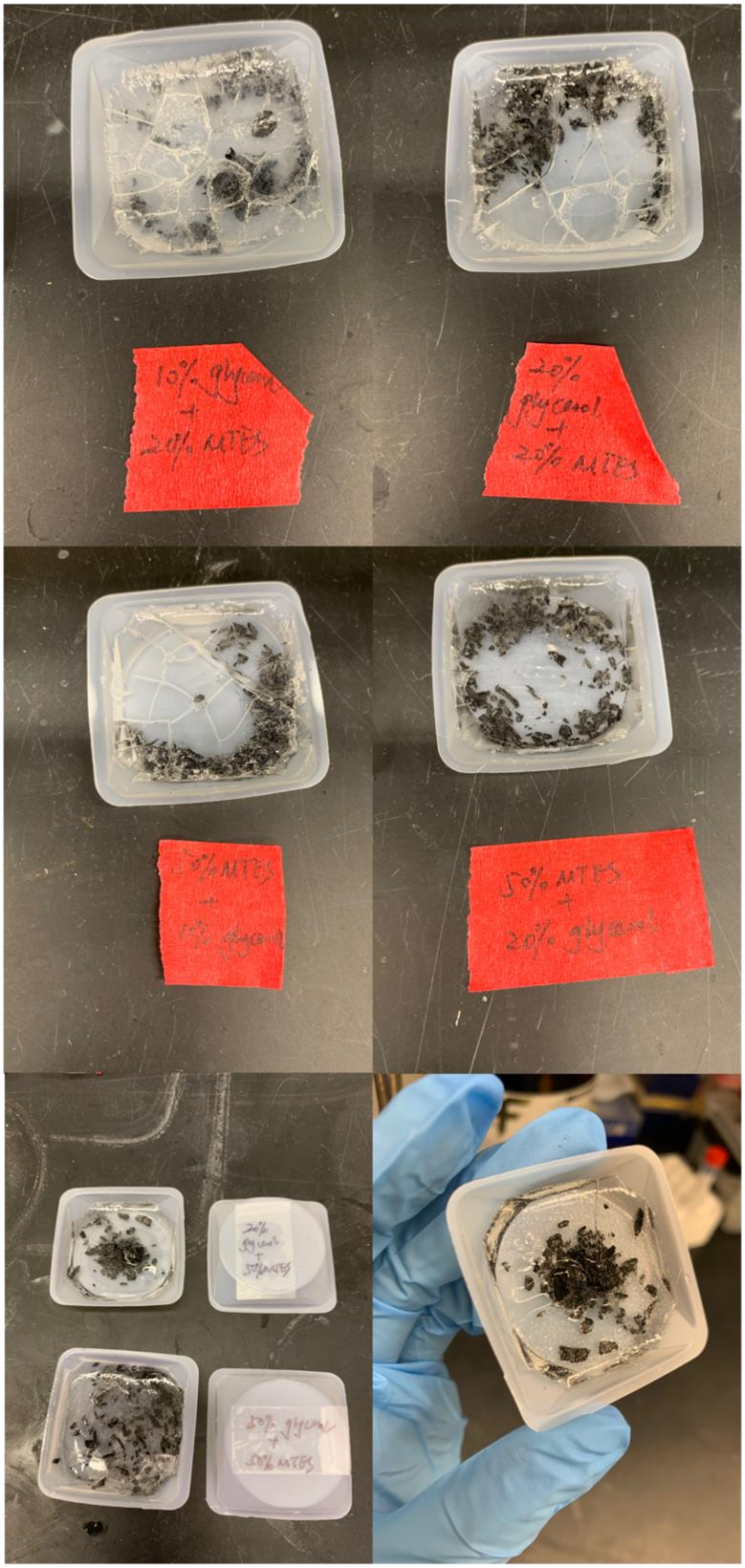

Figure S8. Gel plates with different MTES and glycerol additions.

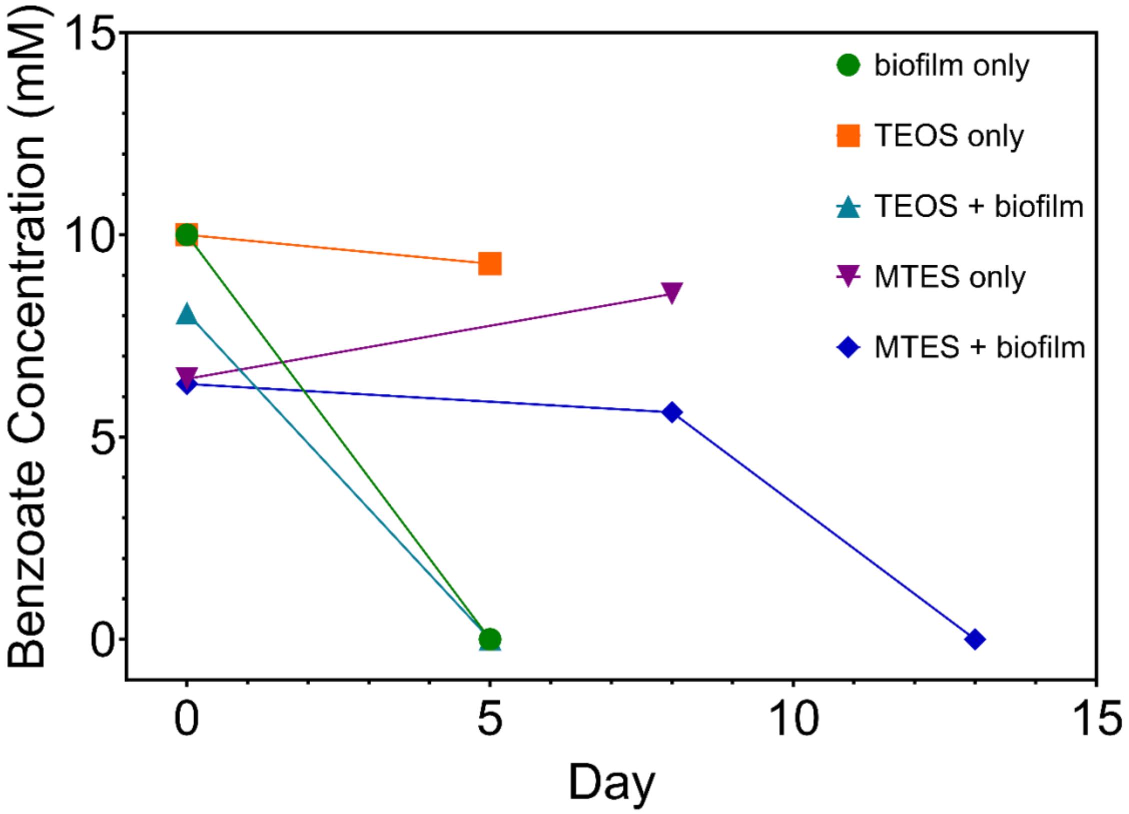

Figure S9. Benzoate degradation over time by various gel plates. Three control groups: “biofilm only”, “TEOS only”, and “MTES only”. “Biofilm only” represented that no sol-gel coating was applied but only biofilm-SB (corn kernel biochar) was added to test the benzoate degradation capability. “TEOS only” and “MTES only” represented that no biomass was added but only sol-gel coating with different recipes, where “TEOS only” gel was made by 21:1 (water:TEOS) and “MTES only” was made by 1:1.55:11.41 (MTES:TEOS:water). Two treatment groups: “TEOS + biofilm” and “MTES + biofilm”. Both treatment groups represented that biofilm-SB was coated by gel plates, and the recipes corresponded to control groups.

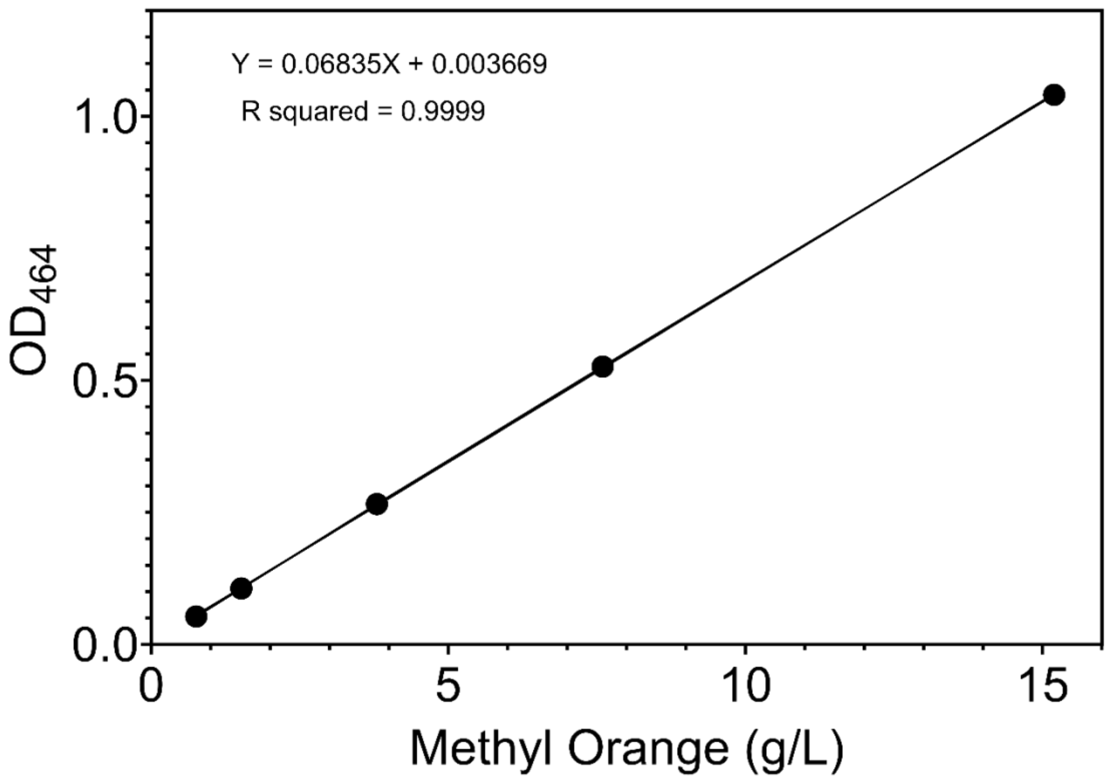

Figure S10. Calibration curve of Methyl Orange concentration over OD_{464}.

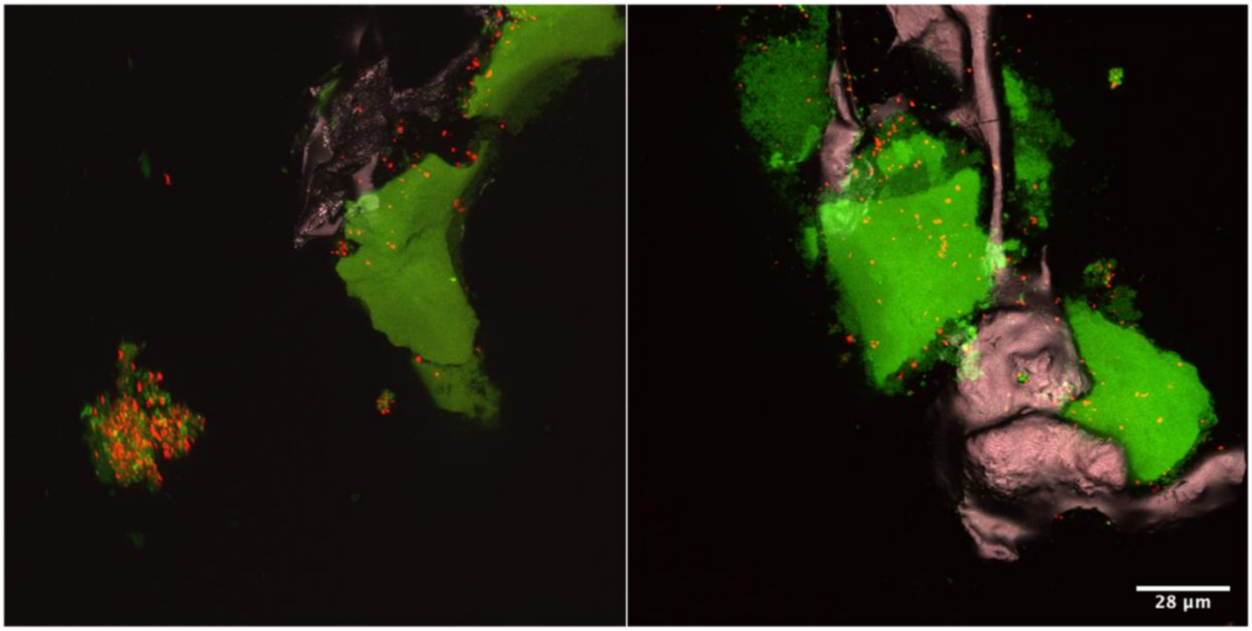

Figure S11. Cell viability of sol-gel encapsulated biofilm-BC after adding only one benzoate spike (10 mM) within three months. Biofilm-BC was coated by recipe-F, where recipe-F: MTES/TEOS/water = 0.5:0.5:20, PVP = 10 mg, PEG400: 20% (v/v), two-time coating (tea ball)(MTDP-P).

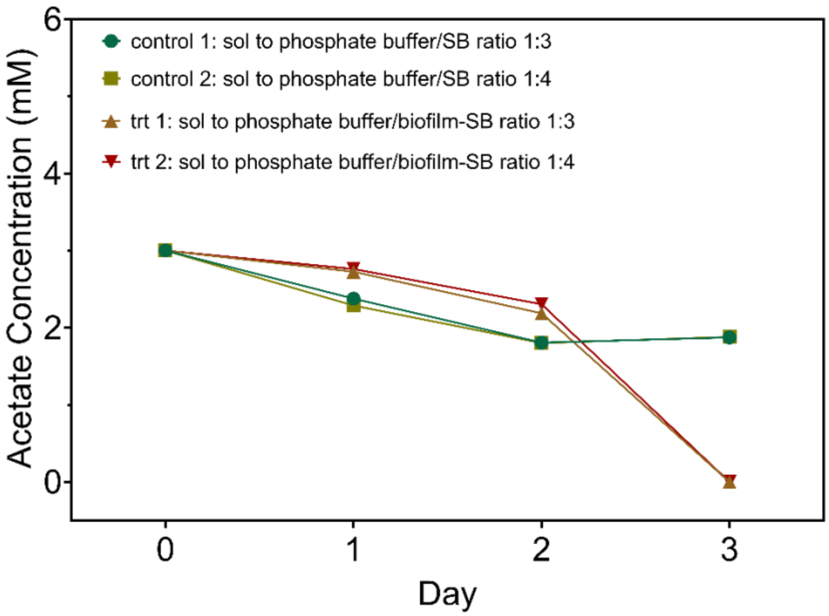

Figure S12. Acetate degradation of LB400 biofilms coated by various sol to phosphate buffer ratios over time. "trt1" and "trt2" were treatment groups which represented that LB400 biofilms were coated by sol to phosphate buffer ratios of 1:3 and 1:4, respectively. "Control 1" and "control 2" were control groups which were made the same way without biomass addition (n=1).

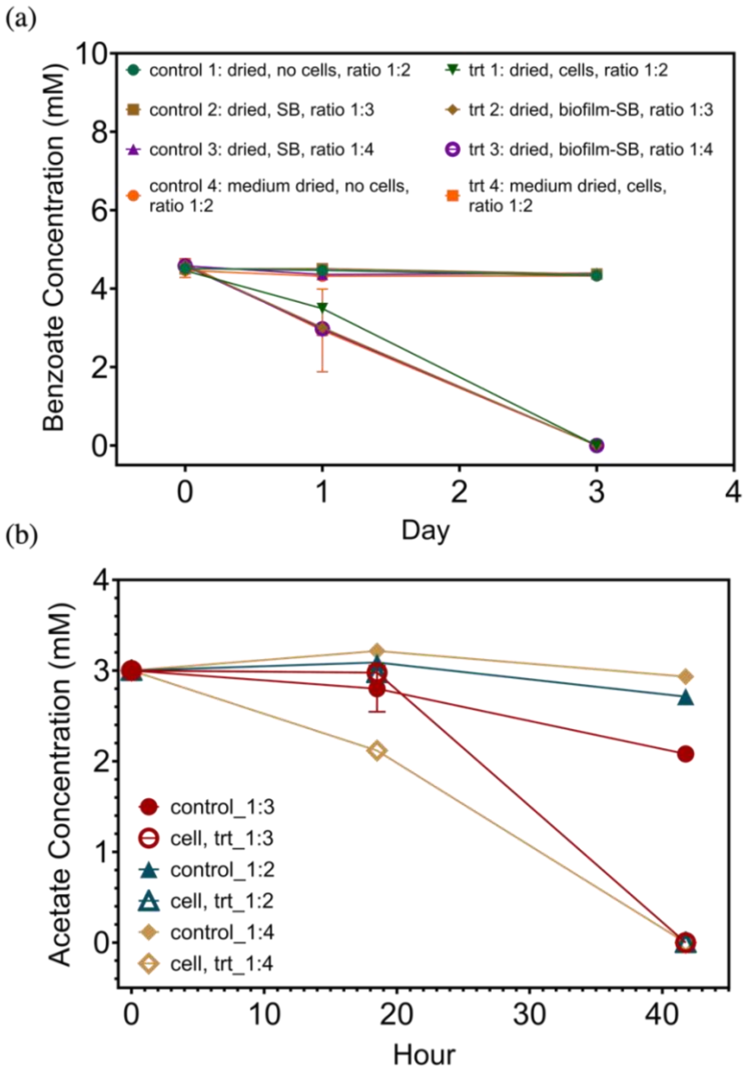

Figure S13. Benzoate and acetate biodegradation of LB400 cells and biofilms coated by various sol to phosphate buffer ratios. (a) Benzoate degradation over time of LB400 cells and biofilms coated by various sol to phosphate buffer ratios. Four treatment groups: “trt 1” represented that LB400 cells were coated by the ratio of 1:2 and then aged until gel was fully dried; “trt 2” represented that LB400 biofilms on SB (corn kernel biochar) were coated by the ratio of 1:3, and then aged until gel was fully dried; “trt 3” represented that LB400 biofilms on SB (corn kernel biochar) were coated by the ratio of 1:4, and then aged until gel was fully dried; “trt 4” represented that LB400 cells were coated by the ratio of 1:2 and then aged for three days. Controls followed the same coating recipe and procedure but had no biomass added (n=2). (b) Acetate degradation of LB400 cells coated by various sol to phosphate buffer ratios. “cell, trt_1:3”, “cell, trt_1:2”, and “cell, trt_1:4” were treatment groups which represented that LB400 cells were coated by corresponding sol to phosphate buffer ratios, while controls were made the same way without biomass addition (n=2). Recipe was 1:20 (TEOS: water) with sol to phosphate buffer ratio ranging from 1:2 to 1:4. The error bars represent the standard errors of biological duplicates.
